# Heparan sulfate proteoglycans undergo differential expression alterations in right sided colorectal cancer, depending on their metastatic character

**DOI:** 10.1186/s12885-015-1724-9

**Published:** 2015-10-20

**Authors:** Iván Fernández-Vega, Olivia García-Suárez, Beatriz García, Ainara Crespo, Aurora Astudillo, Luis M. Quirós

**Affiliations:** 1 Servicio de Patología. Hospital Universitario de Araba, Álava, 01009 Spain; 2 Department of Morphology and Cell Biology, University of Oviedo, 33006 Oviedo, Spain; 3 University Institute of Oncology of Asturias, Oviedo, Spain; 4 Department of Functional Biology, University of Oviedo, 33006 Oviedo, Spain; 5 Department of Biotechnology, Neiker-Tecnalia Arkaute, 01080 Vitoria-Gasteiz, Spain; 6 Department of Pathology, Hospital, Universitario Central de Asturias, 33006 Oviedo, Spain

**Keywords:** Colorectal cancer, Heparan sulfate, Proteoglycan, Glycosaminoglycan, Chondroitin sulfate

## Abstract

**Background:**

Heparan sulfate proteoglycans (HSPGs) are complex molecules involved in the growth, invasion and metastatic properties of cancerous cells. This study analyses the alterations in the expression patterns of these molecules in right sided colorectal cancer (CRC), both metastatic and non-metastatic.

**Methods:**

Twenty right sided CRCs were studied. A transcriptomic approach was used, employing qPCR to analyze both the expression of the enzymes involved in heparan sulfate (HS) chains biosynthesis, as well as the proteoglycan core proteins. Since some of these proteoglycans can also carry chondroitin sulfate (CS) chains, we include the study of the genes involved in the biosynthesis of these glycosaminoglycans. Immunohistochemical techniques were also used to analyze tissue expression of particular genes showing significant expression differences, of potential interest.

**Results:**

Changes in proteoglycan core proteins differ depending on their location; those located intracellularly or in the extracellular matrix show very similar alteration patterns, while those located on the cell surface vary greatly depending on the nature of the tumor: glypicans 1, 3, 6 and betaglycan are affected in the non-metastatic tumors, whereas in the metastatic, only glypican-1 and syndecan-1 are modified, the latter showing opposing alterations in levels of RNA and of protein, suggesting post-transcriptional regulation in these tumors. Furthermore, in non-metastatic tumors, polymerization of glycosaminoglycan chains is modified, particularly affecting the synthesis of the tetrasaccharide linker and the initiation and elongation of CS chains, HS chains being less affected. Regarding the enzymes responsible for the modificaton of the HS chains, alterations were only found in non-metastatic tumors, affecting N-sulfation and the isoforms HS6ST1, HS3ST3B and HS3ST5. In contrast, synthesis of the CS chains suggests changes in epimerization and sulfation of the C4 and C2 in both types of tumor.

**Conclusions:**

Right sided CRCs show alterations in the expression of HSPGs, including the expression of the cell surface core proteins, many glycosiltransferases and some enzymes that modify the HS chains depending on the metastatic nature of the tumor, resulting more affected in non-metastatic ones. However, matrix proteoglycans and enzymes involved in CS fine structure synthesis are extensively modified independetly of the presence of lymph node metastasis.

**Electronic supplementary material:**

The online version of this article (doi:10.1186/s12885-015-1724-9) contains supplementary material, which is available to authorized users.

## Background

Malignances originating in the gastrointestinal tract include pathologies like esophageal, gastric, pancreatic, hepatocellular or colorectal cancer which are responsible for about one third of all cases of cancer in the world [1]. Cancer of the colon and rectum are often combined and referred to as colorectal cancer (CRC). CRC is the third most common cancer in both men and women, and the third leading cause of cancer-related death in the world [1]. About 90–95 % of CRCs arise sporadically, and different intrinsic and extrinsic factors are associated with increased risk of developing these tumors [2]. The remaining 5 to 10 % are due to inherited conditions, including familial adenomatous polyposis and hereditary non-polyposis CRC [2].

Although in general CRCs are usually adenocarcinomas, arising from the normal mucosa of the large bowel and are histologically similar in appearances, this is in fact a heterogeneous disease when considered with respect to the anatomic location of the tumor, and there are anatomic, functional and molecular differences between tumors of the proximal and distal colorectum, including differences in the phenotypic expression of various biomarkers, as well as the fact that they usually differ in their response to screening tests, the stage at which they are diagnosed, their etiology and furthermore, their effect on mortality [2]. Microsatellite instability is more common in right sided cancers, and these tumors usually have an improved prognosis. In contrast, left sided tumors often display features, such as mutation in p53 or overexpression of VEGF, that are associated with poor prognosis [2, 3].

Few advances have been made in the description of the molecular changes involved in the invasive stages of CRC tumors; however, various molecular phenotypes have been associated with aggressive subtypes in these pathologies [2]. Notably, several of these markers show a relationship with the heparan sulfate proteoglycans (HSPGs). As such, p53 has been described to be involved in the regulation of genes such as SULF2 or heparanase [4, 5]; MUC1 and HSPGs cooperate in cell-cell dissociation and the invasiveness of colon carcinoma cells [6]; many members of the TGF-beta cytokine superfamily bind to heparan sulfate (HS) chains [7]; the Wnt/β-catenin pathway is regulated by glypican-3 and—4 [8, 9]; and HSPGs regulate the activity of VEGF, EGF, and other markers related with CRC [10, 11]. Moreover, recent analysis of trancriptomic profiles of CRCs representing different stages of tumorigenesis have identified important changes in various molecular pathways during tumor development and, interestingly, many of the downregulated genes encode proteins involved in early-phase signal transduction [12].

HSPGs comprise a reduced and specific group of proteins which are covalently linked to HS glycosaminoglycan (GAG) chains. HS is a complex biopolymer, initially created as a chain of alternating D-glucuronic acid (GlcA) and N-acetyl-D-glucosamine (GlcNAc). At various positions, the molecule is modified by a series of interdependent enzymatic reactions that include N-deacetylation of GlcNAc, usually followed by N-sulfation to produce GlcNSO_3_, thus creating sulfated S-domains. Within these regions, GlcA may be epimerized to iduronate (IdoA), and O-sulfate groups added at C6 of GlcN and C2 of IdoA residues. Minor sulfations at C3 of GlcN and C2 of GlcA may also occur. These chain modifications result in clusters of flexible, highly sulfated IdoA-rich regions, separated by more rigid lowly or non-sulfated regions [13, 14]. HSPGs are ubiquitously present in tissues, mainly associated with the cell surface and the extracellular matrix (ECM) [13, 14], and a variety of both normal and pathological functions have been ascribed to them, including cell adhesion and migration, organization of the ECM, regulation of proliferation, differentiation and morphogenesis, cytoskeleton organization, tissue repair, inflammation, vascularization and cancer metastasis; the function ultimately depending on the fine structure of the chains [13–16]. Specific sets of variably modified disaccharides, usually within the sulfated domains, define binding sites for a multitude of specific ligands such as cytokines, chemokines, growth factors, enzymes and enzyme inhibitors, and ECM proteins [14, 15]. Cells exercise exquisite control over both HSPG composition and sequence, though this varies between cell types, developmental stages, and also as a result of cell transformation in pathological processes. It is therefore of interest to analyze in detail the complete range of changes in the expression of HSPGs and HS biosynthetic enzymes occurring in cancer pathologies as well as the effect of these specific signatures on promoting invasion and metastasis.

In several cancer cells, genes involved in the biosynthesis of HSPGs are either up—or downregulated [17]. Some alterations in expressions of colon cancer HSPGs have been described, including alterations which affect the core proteins, as is the case of some syndecans [18], the relative amounts and structure of the GAGs [19, 20], or the expression levels of certain enzymes responsible for the structure of HS saccharidic chains, including glycosyltransferases as EXTL3 [21] or sulfotransferases as 3-OST-2 [22]. However, to date no studies have analyzed the entire set of genes involved in the synthesis of these molecules in this pathology.

In this paper, we investigate the expression patterns of all genes involved in HSPG biosynthesis in CRCs, and compare them with healthy tissues from the same patients. Although previous studies have analyzed this pathology in a general way, here we take into consideration that these tumors are, as already mentioned, heterogeneous in terms of their anatomic location, and focus our study on right sided malignances. The tumors were subdivided into two groups depending on the presence or absence of metastases in lymph nodes, since this element is a key predictor of progression. The study includes genes coding for HSPG core proteins and HS chain synthesis and modification. Taking into account that some of these PGs can also carry chains of chondroitin sulfate (CS), we extended the study to those genes involved in the biosynthesis of this GAG. The aim of the work is to increase our knowledge of the structural alterations of HSPGs in CRCs, which could in the future prove useful in developing new chemical biology approaches to the retardation of tumor progression by modulating deregulated biosynthetic pathways.

## Methods

### Materials

The following materials were purchased from the manufacturers indicated: RNeasy Kit and RNase-Free DNase from Qiagen (Hilden, Germany); High-Capacity cDNA Reverse Transcription Kit and PowerSYBR Green PCR Master Mix from Applied Biosystems (Foster City, CA); GenElute PCR clean-up kit and 3–3′ diaminobenzidine from Sigma-Aldrich (St. Louis, MO); Biotin 3′ End DNA Labeling Kit from Thermo Scientific (Waltham, MA); In Situ Hybridization Detection System For Biotinylated Probes, EnVision™ G|2 Doublestain System and Envision FLEX target retrieval solution of high pH from Dako (Glostrup, Denmark);. All other chemicals were obtained from commercial sources and were of analytical grade.

The following antibodies were used in this study: Goat Anti-heparanase 1 (L-19), rabbit anti-perlecan (H-300), Goat anti-C4ST-2 (D-20), Rabbit anti-XylT-I (H-74), all these polyclonal antibodies, as well as Mouse anti-NDST1 (FF-2) monoclonal antibody being purchased from Santa Cruz Biotechnology, Inc (Santa Cruz, CA). Rabbit anti-glypican-1 and Rabbit anti-UST, both polyclonal antibodies, and Mouse anti-endostatin monoclonal antibody were from Thermo Scientific (Waltham, MA). Mouse anti-CS (CS-56) monoclonal antibody was from Sigma-Aldrich (St. Louis, MO), Rabbit anti-heparanase-2 polyclonal antibody from GeneTex (Atlanta, GA), mouse monoclonal anti-syndecan-1 (CD138), mouse monoclonal anti-CD34 and rabbit polyclonal anti-CD117 all from DakoCytomation (Carpinteria, CA). Anti-mouse (sc-2020), anti-rabbit (sc-2004) and anti-goat (sc-2005) secondary antibodies were also from Santa Cruz Biotechnology (Santa Cruz, CA).

### Tissue samples

All the samples used in this study were obtained from the Tumor Bank at the Institute of Oncology of Asturias (Asturias, Spain). All the patients were male, and twenty of the samples were from CRCs while the remaining twenty were from the corresponding surrounding healthy tissue from the same patients and were used as control. Diagnoses were evaluated using hematoxylin-eosin-stained slides of all samples according to World Health Organization (WHO) criteria and the snap frozen tissues were stored at −80 °C prior to isolation of the RNA. Applying the TNM classification, all tumors were at the T3 stage (muscularis propria affected) and they were classified into two groups depending on the presence (at least N1) or absence (N0) of lymph node metastases, resulting in 10 samples being included in the first group and 10 in the second. The study was approved by the Ethics Committee on Clinical Investigation of the Hospital Universitario Central de Asturias and written informed consents from the patients were obtained.

### Total RNA isolation and cDNA synthesis

To obtain the RNA, fragments of tissue of between 20 and 30 mg in weight were used. Samples were homogenized using a polytron PT 2100 (Kinematica Inc; Bohemia, NY), and RNA was isolated using the RNeasy kit, following the manufacturer’s specifications. To ensure removal of residual contaminating DNA, samples were subjected to treatment with RNase-free DNase during the purification process itself. The concentration of RNA obtained was determined spectrophotometrically by measuring absorbance at 260 nm of a 1:50 dilution using a BioPhotometer (Eppendorf; Hamburg, Germany). The samples were divided into aliquots of 10 μl and used for reverse transcription reactions or stored at −20 °C until further use.

cDNA synthesis was carried out using the High Capacity cDNA Transcription Kit following the manufacturer’s specifications. The reactions were performed using a thermocycler iCycler IQ (BioRad; Hercules, CA), using 2 μg of RNA as starting material. The reaction products were cleaned using the PCR Clean-Up GenElute kit in line with the manufacturer’s instructions. Finally, the aliquots containing the cDNA were diluted 1:20 with water and used for qRT-PCR assays or stored at −20 °C until use.

### qRT-PCR reactions

In all cases, specific oligonucleotides were designed on different exons or exon junctions, using the program Primer 3. (http://biotools.umassmed.edu/bioapps/primer3_www.cgi). The size of the amplicon in all cases was between 70 and 150 base pairs, ensuring wherever possible that the Tm was above 77 °C. The theoretical Tm for each amplicon was determined using the program Biomath (http://www.promega.com/a/apps/biomath/?calc=tm.). Primer sequences are presented in Additional file 1.

At least four repetitions of all the qRT-PCR reactions were carried out in a final volume of 10 μl, according to the manufacturer’s specifications, using 1 μl of the cDNA dilution as template, with 2 μl of primer pair mix (200 nM final concentration) and 5 μl of SYBR Green mix, assembled in 96 well microtiter plates. The plates were sealed with optical film and centrifuged at 2500 rpm for 5 min before being placed in a Real-Time ABI Prism Detection System device (Applied Biosystems; Foster City, CA) and the following cycling conditions imposed: 95 °C for 10 min, 40 cycles of 95 °C for 15 s followed by 60 °C for 60 s. Following the thermal cycling and data collection steps, amplimer products were analyzed using a melt curve program (95 °C for 1 min, 55 °C for 1 min, then increasing by 0.5 °C per cycle for 80 cycles of 10 s each). For each amplification the presence of a single peak with a Tm corresponding to that previously calculated was verified. Actin was included on each plate as a control gene to compare run variation and to normalize individual gene expression.

### Data analysis

To calculate the efficiencies of amplification for each gene we used the program LinRegPCR (http://www.hartfaalcentrum.nl/index.php?main=files&fileName=LinRegPCR.zip&sub=LinRegPCR), using the best correlation coefficient (considering a minimum of 3 points within the window of linearity) and establishing the average of all positive amplifications. At least 4 replicates of each reaction were carried out, with the number of replicates being increased in those reactions that showed ambiguity or dispersion of results. The values of differential expression of the genes of interest were expressed as has been described previously [23]. A non parametric Wilcoxon test was used for the statistical analysis of the experiments, using a level of significance of *p <* 0.05. All analyses were performed using the program Statistica for Windows (Statsoft Inc; Tulsa, OK).

### Riboprobe preparation

Specific sense and antisense riboprobes for syndecan-1 were designed. The riboprobe sequences were: sense 5′-GAGCCTGCAGCCGGCCCTGCCGCAAATTGTGGCTAC-3′, antisense 5′-GTAGCCACAATTTGCGGCAGGGCCGGCTGCAGGCTC-3′;

The length of the probes was adjusted to between 34 and 36 nucleotides, the content of G + C to between 48 % and 62 % and Tm was always above 75 °C. The probes were labeled with Biotin kit 3 ′End DNA Labeling Kit according to the manufacturer’s specifications.

### Chromogenic *in situ* hybridization (CISH)

To perform the hybridizations, paraffin embedded tissue sections were treated with xylene to render them diaphanous, the paraffin later being removed by passing it through decreasing alcohol concentrations until water was reached. The samples were then incubated at pH 9 in DAKO K8005 buffer for 30 min at 90 °C to facilitate the exposure of cellular RNA. Subsequently, the preparations were washed with sterile tris-buffered saline (TBS), and incubated with labeled probes at a dilution of 1:2.5 in sterile water in a DAKO hybridization oven for 5 min at 95 °C, followed by 15.5 h at 62 °C. Then, the preparations were washed with TBS for 10 min, followed by a second 5 min wash. The entire procedure was carried out using the In Situ Hybridization Detection System for Biotinylated Probes according to the manufacturer’s specifications. Sections were fixed, mounted and examined with a Leica DMR microscope (Wetzlar, Germany). Visualization was carried out using a DFC295 Leica camera.

### Immunohistochemistry

Tissue sections were dewaxed as described in the previous section. Rehydrated sections were rinsed in phosphate buffered saline (PBS) containing 1 % tween-20. Sections were heated in high pH Envision FLEX target retrieval solution at 65 °C for 20 min and then incubated for 20 min at room temperature in the same solution. Endogenous peroxidase activity (3 % H_2_O_2_) and non-specific binding (33 % fetal calf serum) were blocked and the sections were incubated overnight at 4 °C with primary antibodies using a 1:100 dilution. Secondary antibodies were used at a 1:100 dilution. 3–3′ diaminobenzidine was used as a chromogen. Finally, samples were counterstained with hematoxylin, dehydrated and mounted in Entellan® (Merck, Germany). The sections were studied and photographed (20X and 40x objective) under a light microscope (Nikon—Eclipse 80i) (Nikon Corporation, Tokio).

## Results

### Analysis of differential gene expression

We investigated the differential expression of practically all the known genes involved in the defined steps of the biosynthesis of HSPGs in CRCs. We divided the sample into two groups taking the presence or absence of metastases in lymph nodes as the key indicator. 10 samples were obtained from patients lacking metastases; their mean age was 65 ± 16 years; histological grade in all cases was moderate; the average tumor size was 5.5 ± 1.8 x 3.9 ± 1.8 cm. In addition, 10 samples were obtained from patients who showed lymph node metastases in 100 % of cases, their mean age was 69 ± 8 years; histological grade was moderate; the average tumor size was 4,7 ± 0,9 x 4.4 ± 1,7 cm.

We used qRT-PCR to perform a quantitative analysis of mRNA expression. In many of the genes in which we were able to detect differences between normal tissues and tumors, we complemented this test by determining the expression using histological techniques, including *in situ* hybridizations and immunohistochemistry.

### Differential expression of genes encoding core proteins carrying HS chains

**Fig. 1:**
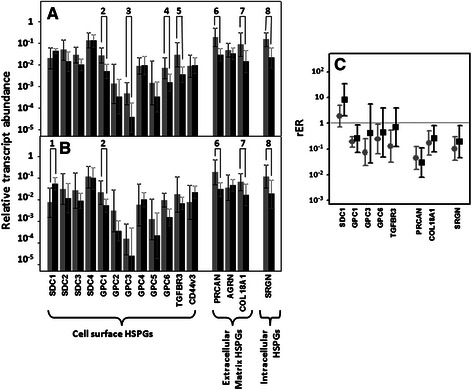
Differential transcription of genes encoding HSPGs. **a**, **b** Relative transcript abundance of mRNAs for HSPGs, grouped according to their topographic location: on the cell surface, in the extracellular matrix or intracellular. Relative abundance for healthy tissues (gray bars) and tumors (black bars) are plotted on a log scale for each gene assayed and the spreads represent the standard deviations. Genes that display significant differences in their transcription levels are highlighted. **a** Non-metastatic CRCs. 2: glypican-1 (*p <* 0.01); 3: glypican-3 (*p* = 0.018); 4: glypican-6 (*p* = 0.02); 5: betaglycan (*p <* 0.01); 6: perlecan (*p* = 0.01); 7: collagen XVIII (*p* < 0.01); 8: serglycin (*p* = 0.01). **b** Metastatic CRCs. 1: syndecan-1 (*p* = 0.02); 2: glypican-1 (*p* = 0.03); 6: perlecan (*p* = 0.015); 7: collagen XVIII (*p* = 0.04); 8: serglycin (*p* = 0.017). **c** Relative expression ratio of genes that show statistically significant differences in expression in non-metastatic (●) or metastatic (■) CRCs. Values on the Y axis are represented on a logarithmic scale

**Fig. 2:**
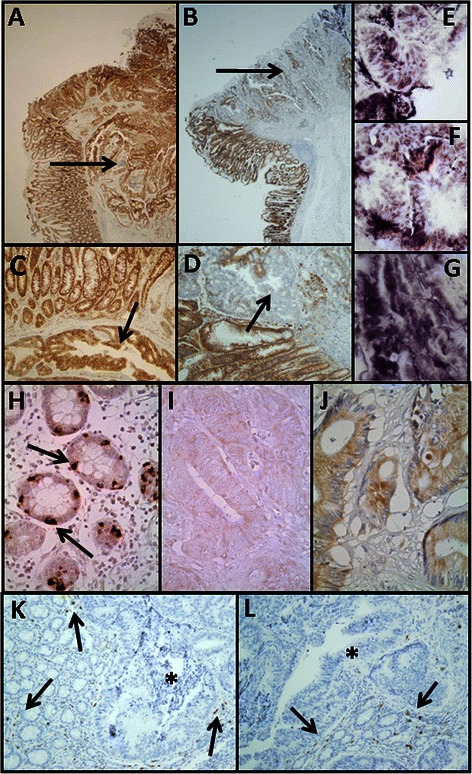
Immunolocalization of cell HSPGs. **a**-**g** Histological localization of syndecan-1 expression. **a**-**d** Localization of syndecan-1 in transition area between normal colon mucosa and tumor using immunohistochemistry. **a**, **c** Non-metastatic tumor; arrows indicate the retention of staining in the tumor region; **b**, **d** Metastatic tumor; arrows indicate the loss of staining in the tumor region, magnification A and B 20X, magnification C and D 100X; **e**-**g** CISH for syndecan-1 in CRCs. **e** Normal tissue; **f** Non-metastatic tumor; **g** Metastatic tumor; magnification 400X. **h**-**j**. Immunolocalization of glypican-1 expression. **h**, Normal mucosa. Intense staining appears in cells with neuroendocrine differentiation (arrows), whereas other epithelial cells show weak to moderate staining. **i** Non-metastatic CRC showing extremely weak staining. **J** Metastatic tumor showing weak staining levels; magnification 400X. **k**-**l** Immunolocalization of mast cells using CD117 antibody. Localization of mast cells in transition area between normal colon mucosa and tumor. **k** Non-metastatic CRC; **l** Metastatic CRC; magnification 200X. Arrows indicate the staining of mast cells in the normal mucosa. The asterisks show the tumor area, in which there is no detectable labeling

HS occurs in cells and the ECM as PGs [24]. Two gene families, syndecans and glypicans, account for most cell surface HSPGs, each of which consists of 4 and 6 isoforms, respectively (*SDC1-4* and *GPC1-6*). Within the syndecans group, no significant differences in the levels of transcripts of isoforms 2, 3 and 4 could be detected in any tumor group, regardless of the presence or absence of lymph node metastasis (Fig. 1a and b). However, syndecan-1 mRNA appeared overexpressed approximately 8 fold in metastatic tumors only (*p* = 0.02, Fig. 1c), the overexpression occurring in 90 % of the metastatic CRCs analyzed. Changes in syndecan-1 were also evaluated immunohistochemically using monoclonal anti-SDC1. Metastatic tumors showed appreciable levels of immunoreactivity, a similar intensity to that detected in the normal tissues from the same patients (Fig. 2a and c). In contrast, and surprisingly, the metastatic tumors showed a dramatic decrease in staining, which largely disappeared, while labeling was nitidus in the adjacent normal mucosa (Fig. 2b and d). The divergence between the results obtained for the quantification of mRNA levels by qRT-PCR and the immunohistochemical analysis of the protein suggested the value of using CISH to clarify the situation. These was done using biotinylated probes for *SDC1*, which showed a positive hybridization in normal and tumoral cells, both metastatic and non-metastatic, consistent with the differential transcription data obtained by qRT-PCR (Fig. 2e, f and g).

Analysis of the expression levels of the different glypicans revealed the presence of transcripts for all of the 6 different isoforms, although their levels varied widely depending on the particular species examined. We could not detect statistically significant differences for isoforms 2, 4 and 5, associated with tumor transformation. GPC1 was the only isoform that appeared underexpressed in both metastatic and non-metastatic tumors (Fig. 1a and b); in metastatic tumors, its levels were reduced in 80 % of the cases analyzed (*p* = 0.03), being around 25 % of those determined in healthy tissues (Fig. 1c); meanwhile, in non metastatic tumors, its levels were reduced in all cases analyzed (*p* < 0.01), and their values were around 18 % of those determined in healthy tissues (Fig. 1c). Analysis of protein expression by immunohistochemistry revealed the existence of strong staining in the epithelial cells, although only in isolated cells displaying neuroendocrine differentiation (Fig. 2h). Immunostaining dropped dramatically in the tumor samples, particularly in non-metastatic tumors (Fig. 2i and J).

In addition to GPC1, decreases in the levels of transcription of glypicans 3 and 6 were also detected, although only in non-metastatic tumors (Fig. 1). GPC3 appeared underexpressed in 100 % of non-metastatic CRCs analyzed (*p* = 0.018), with the average decrease being approximately 14 fold (Fig. 1c), while only 50 % of metastatic tumors showed alteration in levels of this glypican. Meanwhile, GPC6 mRNA levels were reduced almost 4 fold in 80 % of non-metastatic tumors (*p* = 0.02), a percentage that halved in the case of tumors with lymph node metastases (Fig. 1c).

Beside syndecans and glypicans, the other HSPGs present in the cell membrane include betaglycan (TGFBR3) and CD44v3 isoform [24]. qRT-PCR analysis showed the presence of transcripts of these genes in normal tissues and in tumors, both metastatic and non-metastatic (Fig. 1a and b). We were unable to determine significant differences in their transcript levels, except for betaglycan in non-metastatic tumors, where it appeared underexpressed in 90 % of cases (*p <* 0.01), with values around 8 times lower than those of normal tissues (Fig. 1c).

Serglycin is a cell-associated PG that differs from cell surface HSPGs in that it is located intracellularly [25]. qRT-PCR analysis of serglycin transcripts in non-metastatic CRCs showed a decrease in 100 % of the tumors investigated (*p* = 0.01), the average reduction being around 10 fold (Fig. 1). When the study was performed on metastatic CRCs, a decrease in RNA levels affecting 70 % of the tumors was also detected (*p* = 0.017), though the average reduction was half of that observed in the non-metastatics (Fig. 1). The principal intracellular locations of serglycin are mast cell secretory granules [25], prompting us to apply immunohistochemical studies to detect these cells using the antibody CD117. The analysis showed a drastic reduction of the population of mast cells in tumors compared to non-tumor colon mucosa (Fig. 2k and l).

**Fig. 3:**
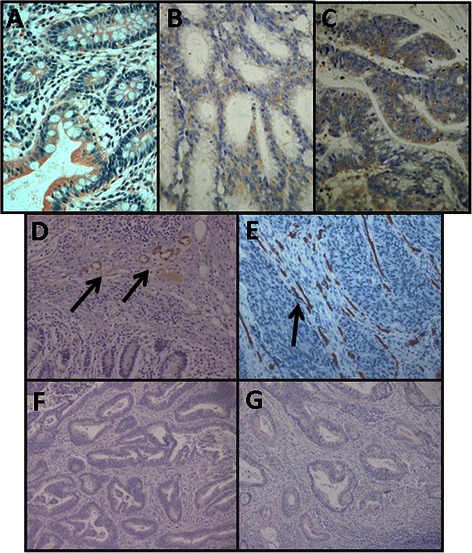
Immunolocalization of ECM HSPGs. Immunolocalization of perlecan expression. **a** Normal mucosa showing positive staining in the cell cytoplasm and in the basement membranes, magnification 400X. **b**, **c** Non-metastatic (**b**) and metastatic (**c**) tumors showing weak and focal staining in the cells and the tumor stroma; magnification 400X. **d**-**g** Immunolocalization of collagen XVIII expression. **d** Normal mucosa. Staining appears at arteriolar vessels in the lamina propria (arrows). **f**, **g** Non-metastatic (**f**) and metastatic (**g**) CRCs showing no positive immunoreactivity. **e** Tumor neovascularization displayed by positive immunoreactivity for CD34 antibody. Magnification 200X

When evaluating the extracellular matrix PGs, no significant differences were detected for agrin. However, perlecan and collagen XVIII showed significant down regulations in tumoral samples. The transcription levels of perlecan decreased in 90 % of both non-metastatic and metastatic tumors (*p* = 0.01 and 0.015), respectively reaching values around 20 and 30 fold lower than those of healthy tissues. These results were also observed using immunohistochemistry, where perlecan displayed only a faint staining of the cytoplasm and basement membranes of normal tissues, while weaker staining was observed in the tumor stroma of metastatic and non-metastatic CRCs (Fig. 3a, b and c). Moreover, a significant subexpression of collagen XVIII was also detected, around 6 and 4 times lower than in normal tissue for metastatic and non-metastatic tumors respectively (*p <* 0.01 and *p* = 0.04) and was observed in all cases of non-metastatic CRCs analyzed and in 70 % of metastatic. Immunohistochemical analysis revealed that healthy tissues expressed the protein mainly at arteriolar vessels in the lamina propria (Fig. 3d); in contrast, the expression of the protein was not observed in the tumors, although capillary neovascularization was able to be detected, evidenced by immunostaining using the anti-CD34 antibody (Fig. 3e, f and g).

### Differential expression of genes encoding glycosyltransferases involved in common linkage region sequence and GAG chain synthesis

HS and CS/dermatan sulfate (DS) chains are synthesized through the cooperation of multiple biosynthetic enzymes in the Golgi. The first step requires an array of glycosyltransferases (GTs) which generate a tetrasaccharide gycan linker on a cognate serine residue of the PG core. This linker is shared by HS and CS/DS chains and its sequence is integrated by xylose-galactose-galactose-GlcA [25, 26]. HS chain extension requires the subsequent transfer of a GlcNAc residue, followed by the sequential addition of alternative GlcA and GlcNAc residues to generate a non-branched polymer. In contrast, the addition of an N-acetyl-D-galactosamine (GalNAc) rather than GlcNAc directs the pathway towards the biosynthesis of CS/DS. In this case, chain extension takes place through the sequential addition of alternative GlcA and GalNAc residues [26, 27].

**Fig. 4:**
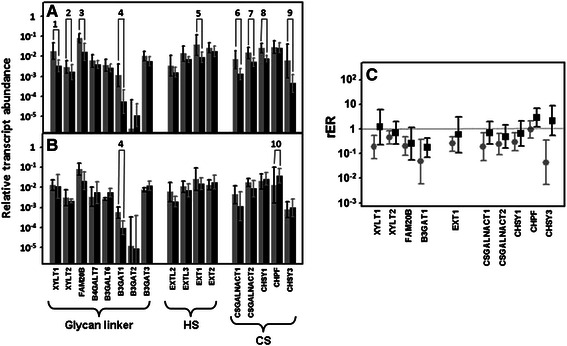
Differential transcription of genes encoding the glycosyltransferases involved in the biosynthesis of HS and CS chains. **a**, **b** Relative transcript abundance of mRNAs for enzymes involved in the synthesis of the tetrasaccharide gycan linker, the initiation and elongation of HS chains, and the initiation and elongation of CS chains. Relative abundance for healthy tissues (gray bars) and tumors (black bars) are plotted on a log scale for each gene assayed and the spreads represent standard deviations. Genes that display significant differences in their transcription levels are highlighted. **a** Non-metastatic CRCs. 1: xylosyltransferase I (*p* < 0.01); 2: xylosyltransferase II (*p* = 0.017); 3: xylosylkinase (*p* = 0.017); 4: beta-1,3-glucuronyltransferase 1 (*p* = 0.018); 5: exostosin glycosyltransferase 1 (*p* = 0.01); 6: chondroitin sulfate N-acetylgalactosaminyltransferase 1 (*p* = 0.018); 7: chondroitin sulfate N-acetylgalactosaminyltransferase 2 (*p* = 0.011); 8: chondroitin sulfate synthase 1 (*p* = 0.01); 9: chondroitin sulfate synthase 3 (*p* = 0.01). **b** Metastatic CRCs. 4: beta-1,3-glucuronyltransferase 1 (*p* = 0.018); 10: chondroitin polymerizing factor (*p* = 0.043). **c** Relative expression ratio of genes that showed statistically significant differences in expression in non-metastatic (●) or metastatic (■) CRCs. Values on the Y axis are represented on a logarithmic scale

**Fig. 5:**
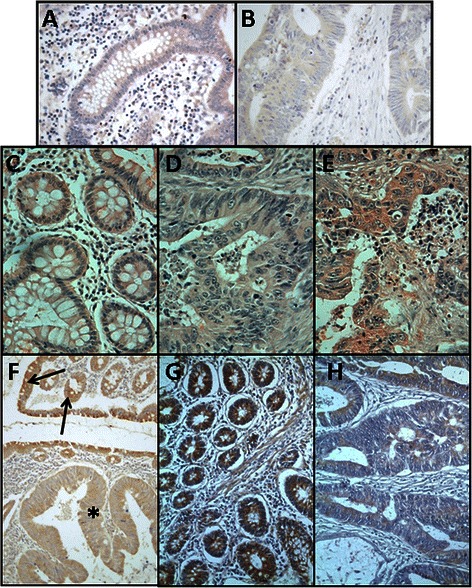
Immunolocalization of genes involved in the biosynthesis of HS and CS chains. **a**, **b** Immunolocalization of XYLT1 expression. **a** Normal mucosa showing weak to moderate staining. **b** Non-metastatic CRC, in which there is no detectable labeling; magnification 400X. **c**-**e** Immunolocalization of NDST1 expression. **c** Normal mucosa showing weak to moderate staining in the cell cytoplasm. **d**, Non-metastatic CRCs showing a decrease in the intesity of the staining. **e** Metastatic CRCs showing a similar staining intensity to that of healthy tissue. Magnification 400X **f** Localization of CHST12 expression in transition area between normal colon mucosa and tumor. Arrows indicate the staining of cells in the normal mucosa. The asterisks show the tumor area, in which the intensity of the labeling diminishes. Magnification 400X. **g**, **h** Immunostaining of UST. **g** Normal mucosa showing cytoplasmic staining in epithelial cells. **h** Non-metastatic CRC showing a notable decreasing in labeling. Magnification 400X

We analyzed the differential transcription of the various genes known to be involved in the biosynthesis of the tetrasaccharide linker: *XYLT1* and *XYLT2*, which are responsible for the initial transfer of the xylose residue; *FAM20B*, which encodes a xylose kinase that catalyzes the transient phosphorylation of this residue; *B4GALT7*, and *B3GALT6*, responsible for sequential addition of the two residues of galactose; and *B3GAT1*, *B3GAT2* and *B3GAT3*, which encode enzymes responsible for the transfer of GlcA [28]. Four of these transcripts appeared downregulated in non-metastatic CRCs, while only one was in metastatic tumors (Fig. 4). Transcript levels of the three genes involved in the transfer and modification of the xylose residue, *XYLT1*, *XYLT2* and *FAM20B* appeared altered in non-metastatic patients; expression of both *XYLT1* and *FAM20B* decreased by over 80 % in 90 % of patients, while *XYLT2* expression fell by around 60 % in 80 % of analyzed cases (*p* = 0.008, *p* = 0.02 and *p* = 0.017 respectively). Conversely, in metastatic tumors no statistically significant differences in the expression levels of any of these genes were detected (Fig. 4). We carried out an analysis of XYLT1 protein expression using immunohistochemistry which showed the existence of staining in the normal tissues that could not be detected in experiments performed using non-metastatic tumor samples (Fig. 5a and b). No differences in expression were observed for *B4GALT7*, and *B3GALT6*, or for two of the three isoforms involved in the transfer of the first GlcA residue, *B3GAT2* and *B3GAT3*, although *B3GAT2* did display very low expression values which varied depending on the patients analyzed (Fig. 4a and b). Conversely, *B3GAT1* showed a statistically significant subexpression, both in non-metastatic tumors, where its levels were reduced by around 20 fold, and in metastatics, where the decrease was roughly about 6 fold, affecting in both cases at 90 % of patients tested (*p* = 0.018 and *p* = 0.04 respectively).

In no case did the mRNA levels of the GTs involved in the synthesis of the HS chains appear modified in CRCs, neither those enzymes involved in the transfer of the first GlcNAc residue or those in charge of the subsequent polymerization. The only exception being EXT1, which appeared downregulated on average about 4 fold (*p* = 0.01) in 90 % of patients with non-metastatic tumors (Fig. 4).

By contrast, the biosynthesis of CS/DS chains appears to be seriously affected in non-metastatic tumors, as indicated by the subexpression of 4 of the 5 genes involved (Fig. 4a). The two GTs involved in the transfer of the first GalNAc residue, *CSGALNACT1* and *CSGALNACT2,* appeared downregulated an average around 5- and 4 fold in 100 % and 75 % of tumors respectively (*p* = 0.18 and *p* =0.011); in addition, the chondroitin synthases CHSY1 and CHSY3 reduced their mRNA levels about 70 % and over 95 % in 90 % and 70 % of the CRCs analyzed respectively (*p* = 0.01 and *p* =0.05). Interestingly, none of the changes in these transcripts in metastatic tumors were significant, except for *CHPF* (*p* = 0.04), the only gene not altered in non-metastatic tumors which appeared overexpressed around 3 fold (Fig. 4).

### Differential expression of genes involved in the modification of HS chains

**Fig. 6:**
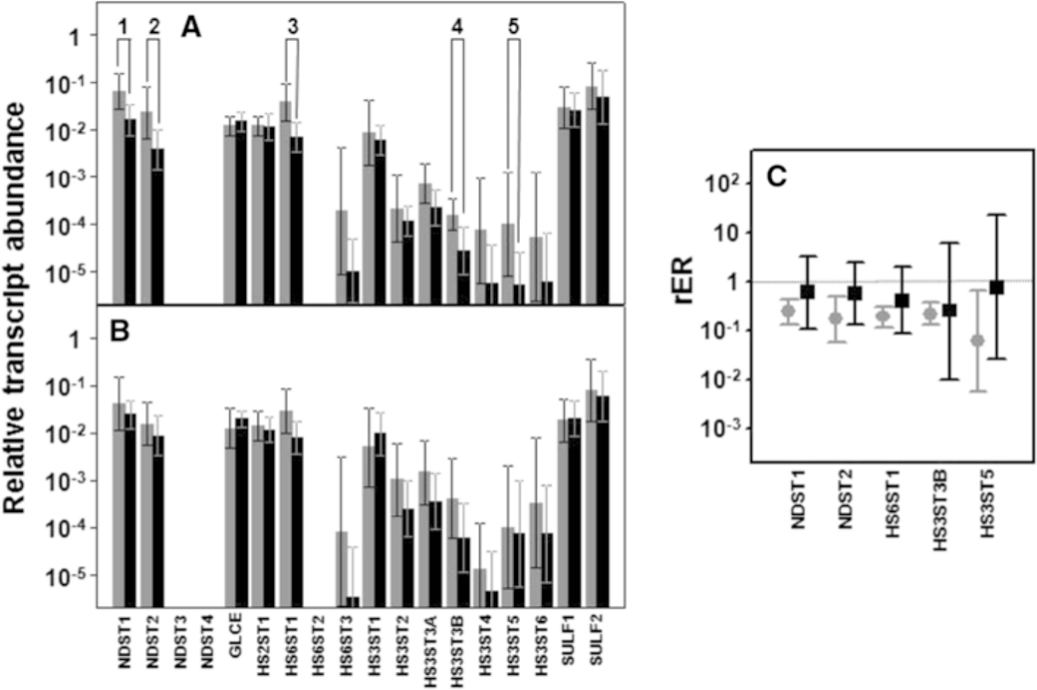
Differential transcription of genes involved in the modification of HS chains. **a**, **b** Relative transcript abundance of mRNAs for enzymes involved in the modification of HS chains. Relative abundance for healthy tissues (gray bars) and tumors (black bars) are plotted on a log scale for each gene assayed and the spreads represent standard deviations. Genes that display significant differences in their transcription levels are highlighted. **a** Non-metastatic CRCs. 1: N-deacetylase/N-sulfotransferase 1 (*p* < 0.008); 2: N-deacetylase/N-sulfotransferase 2 (*p* = 0.008); 3: 6-O-sulfotransferase 1 (*p* = 0.007); 4: 3-O-sulfotransferase 3B (*p* = 0.04); 5: 3-O-sulfotransferase 5 (*p* = 0.009) **b** Metastatic CRCs. **c** Relative expression ratio of genes that show statistically significant differences in expression in non-metastatic (●) or metastatic (■) CRCs. Values on the Y axis are represented on a logarithmic scale

The result of the action of all GTs involved in the synthesis of HS is an unmodified chain that consists simply of repeating GlcNAc-GlcA units. As the chain polymerizes, it undergoes a series of modifications; the initial modification reactions involve removal of acetyl groups from GlcNAc residues, followed by sulfation of the amino group which is catalyzed by four different isoforms of N-deacetylase/N-sulfotransferases: NDST1-4 [13, 14]. Transcripts of only two of these isoforms, *NDST1* and 2, could be quantified in all healthy and tumoral tissues, with *NDST3* and 4 being undetectable in most patients (Fig. 6a and b). Both *NDST1* and 2 appeared downregulated in non-metastatic CRCs alone; *NDST1* showed reduced expression in about 75 % on average (*p* = 0.008), while the reduction in *NDST2* expresion was around 6 fold (*p* = 0.008), the downregulation being observed in both cases in 100 % of tumors analyzed (Fig. 6c). NDST1 expression was also analyzed at the protein level by immunohistochemistry, with similar results to those described for the transcript levels; in normal and metastatic tissues a similar intensity of cytoplasmic staining was observed, while that of non-metastatic CRCs was reduced in comparison (Fig. 5c, d and e).

Further modifications of the HS chain include the epimerization of GlcA into IdoA which is catalyzed by the action of the enzyme C5-GlcA epimerase (GLCE), the addition of sulfate groups at C2 of uronic acid which is catalyzed by the enzyme HS 2-O-sulfotransferase (HS2ST1), and the sulfation at C6 of glucosamine residues, catalyzed by HS 6-O-sulfotransferase isoforms 1–3 (HS6ST1-3) [13, 14]. Neither *GLCE* nor *HS2ST1* showed any significant alterations at the transcriptional level. With regard to C6 sulfation, no *HS6ST2* transcripts could be detected, and the levels of *HS3ST3* were very low and displayed considerable variation between patients; however, *HS6ST1* was the major isoform present in all cases, and furthermore displayed a statistically significant decrease in non-metastatic tumors (*p* = 0.007), values being on average 5 times lower in 100 % of the cases analyzed (Fig. 6).

The final step in the modification of HS chains during their biosynthesis in the Golgi involves the addition of sulfate group at C3 of glucosamine. This reaction is catalyzed by HS 3-O-sulfotransferase isoforms 1–6 (HS3ST1, HS3ST2, HS3ST3A1, HS3ST3B1, HS3ST4, HS3ST5 and HS3ST6) [13, 14]. The transcript levels of these enzymes varied considerably between the different isoforms and also, for some particular isoforms, between the specific cases analyzed. However, it was possible to detect significant differences, confined to non-metastatic tumors, for at least two of these molecules, *HS3ST3B1* and *HS3ST5* (Fig. 6a and b). *HS3ST3B1* decreased around 5 fold in all analyzed CRCs (*p* = 0.004), and *HS3ST5* diminished its transcription levels over 90 % in 80 % of cases (Fig. 6c).

The final modification of the HS patterning is carried out at the cell surface by two cell surface sulfatases, SULF1 and SULF2, which remove glucosamine-6S groups from specific regions [13, 14]. The transcript levels of neither of these genes demonstrated alterations for either type of CRC (Fig. 6).

### Differential expression of genes involved in the modification of CS chains

CS/DS chains are modified to a lesser extent than those of HS. Two reactions may involve the GalNAc residue: sulfation at the C4, catalyzed by four different enzymes (CHST11, CHST12, CHST13 and CHST14), and sulfation at C6, catalyzed by three distinct enzymes (CHST15, CHST3 and CHST7). Another two different reactions may involve the uronic acid residue, including C5 epimerization, catalyzed by dermatan sulfate epimerase (DSE), and the addition of sulfate groups at C2, catalyzed by chondroitin uronosyl sulfotransferase (UST) [28].

**Fig. 7:**
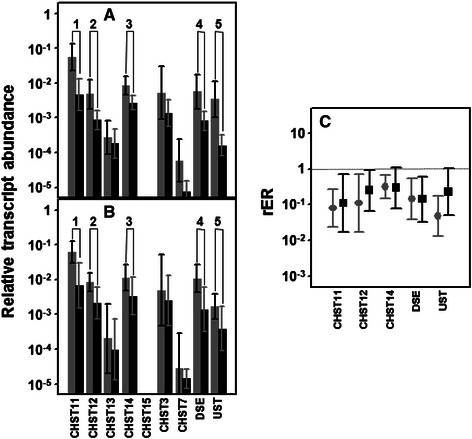
Differential transcription of genes involved in the modification of CS/DS chains. **a**, **b** Relative transcript abundance of mRNAs for enzymes involved in the modification of CS/DS chains. Relative abundance for healthy tissues (gray bars) and tumors (black bars) are plotted on a log scale for each gene assayed and the spreads represent standard deviations. Genes that display significant differences in their transcription levels are highlighted. **a** Non-metastatic CRCs. 1: chondroitin 4 sulfotransferase 11 (*p* = 0.008); 2: chondroitin 4 sulfotransferase 12 (*p* = 0.011); 3: N-acetylgalactosamine 4 sulfotransferase 14 (*p* = 0.012); 4: dermatan sulfate epimerase (*p* = 0.017); 5: uronyl-2-sulfotransferase (*p* = 0.012). **b** Metastatic CRCs. 1: chondroitin 4 sulfotransferase 11 (*p* = 0.02); 2: chondroitin 4 sulfotransferase 12 (*p* = 0.025); 3: N-acetylgalactosamine 4 sulfotransferase 14 (*p* = 0.035); 4: dermatan sulfate epimerase (*p* = 0.046); 5: uronyl-2-sulfotransferase (*p* = 0.05). **c** Relative expression ratio of genes that show statistically significant differences in expression in non-metastatic (●) or metastatic (■) CRCs. Values on the Y axis are represented on a logarithmic scale

Sulfation of GalNAc at C4 seems to be strongly affected in CRCs, since transcription levels of 3 of the 4 enzymes involved appeared downregulated, irrespective of the presence or absence of lymph node metastasis (Fig. 7a and b). In addition, transcript levels of the only gene for which no significant differences could be detected, *CHST13*, were between one and two orders of magnitude lower than the rest. *CHST11* mRNA was the most abundant species in healthy tissues, and its levels were reduced an average of approximately 12 fold in metastatic and 9 fold in non-metastatic tumors (*p* = 0.008 and *p* = 0.021) (Fig. 7c), affecting in both cases 90 % of the patients analyzed. Moreover, *CHST12* showed a 9 fold mean downregulation in the non-metastatic CRCs, in 90 % of cases, whereas in metastatic tumors its decrease was lower, at around 4 fold, and involved only 70 % of cases (*p* = 0.011 and *p* = 0.025). CHST12 protein expression was also analyzed by immunohistochemistry, and the results showed that the staining in the normal colonic mucosa was reduced in tumor tissue (Fig. 5f). Finally, CHST14 showed, on average, a 3 fold deregulation in 80 % of cases for both metastatic and non-metastatic tumors (*p* = 0.012 and *p* = 0.035). However, between the three enzymes involved in C6 sulfation, no significant differences could be detected, albeit that CHST7 mRNA levels were very low and those of CHST15 undetectable (Fig. 7a and b).

Furthermore, the differential transcription levels of the two enzymes involved in the modification reactions of uronic acid residues were significantly altered. DSE decreased around 7 fold, affecting 90 % of non-metastatic and 80 % of metastatic CRCs (*p* = 0.017 and *p* = 0.046) (Fig. 7). Meanwhile, *UST* decreased 25 fold in non-metastatic tumors, affecting all patients tested, although the average decrease in the metastatic tumors was only 4 fold, and in this case only affected 70 % of cases (Fig. 7). The protein expression of UST was also analyzed by immunohistochemistry, staining appearing in the cytoplasm of cells of the normal colonic mucosa, and was greatly decreased in intensity in tumor tissues, consistent with the results at the transcription level (Fig. 5g and h).

**Fig. 8:**
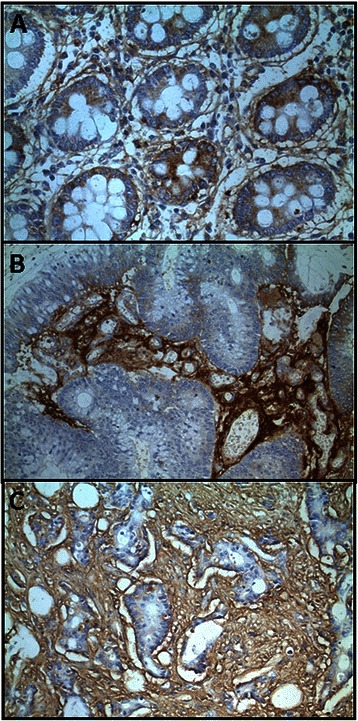
Immunolocalization of CS. **a** Normal mucosa showing stromal and cell labeling. **b** Non-metastatic CRC showing very weak cell labeling and strong stromal staining. **c** Metastatic CRC displaying weak labeling in tumour cells and stroma. Magnification 400X

Given the extent of the observed alterations in the expression of genes involved in the synthesis and modification of CS/DS chains, we also conducted an analysis of the structure of the saccharide chains by immunohistochemistry using the specific antibody CS-56. The results showed a decrease in the intensity of staining in tumor cells, both non-metastatic and metastatic (Fig. 8). However, it was also observed that there was a considerable increase in staining in the stroma of both types of CRC, appearing with greater intensity in the non-metastatics (Fig. 8).

### Differential expression of genes involved in the modification of heparanases

**Fig. 9:**
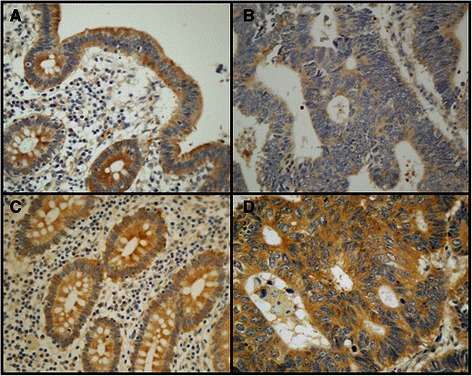
Immunolocalization of heparanases. **a**, **b** Immunostaining of heparanase. **a** Normal mucosa showing intense cell labeling; **b** Non-metastatic CRC from the same patient showing a decrease in immunostaining. **c**, **d** Immunostaining of heparanase 2. Normal mucosa (**c**) and tumor (**d**) samples from the same patient displaying similar immunoreactivity. Magnification 400X

Heparanase (HPSE) is an endo-β-D-glucuronidase that degrades HS, generating biologically active fragments. Analysis of its transcript levels in non-metastatic CRCs established the existence of a statistically significant downregulation (*p* = 0.013). This decrease was observed in 70 % of the patients analyzed, the mean reduction being about 80 % less than in corresponding normal tissues. Interestingly, the dispersion of the results in healthy tissue was much greater than in tumoral tissues, the former exhibiting a standard deviation more than double that of the tumor samples (2.4 versus 1.1 respectively, expressed in cycles of PCR). Furthermore, metastatic tumors showed greater variability in their levels of transcription, *HPSE* mRNA values appearing reduced in 40 % of patients, at the same time as 20 % of patients showing overexpression, results which combined to produce the lack of a statistically significant trend (*p* = 0.13). HPSE expression was also analyzed by immunohistochemistry, where cytoplasmic immunoreactivity was observed in normal tissue cells, the intensity of which decreased in some non-metastatic tumors (Fig. 9a and b).

Heparanase 2 (HPSE2) is a homologue of HPSE which lacks HS-degrading activity, although it is able to interact with HS with high affinity [29]. Transcripts for this gene could be detected in 70 % of both healthy and tumoral tissue of patients with non-metastatic CRCs. Although the difference between tissue types was not statistically significant (*p* = 0.34), as with HPSE, there was much higher dispersion in normal tissue than in tumoral (4.3 versus 1.5 respectively, expressed in cycles of PCR). In metastatic CRCs, these gene transcripts were also detected in 70 % of patients, albeit that values were again highly dispersed. However, the expression could only be detected in 40 % of the tumor samples of this group, with positive expression values being similar to those obtained for non-metastatic tumors. Immunohistochemical analysis of the expression of this protein showed positive immunoreactivity which was of similar intensity in the cells of normal mucosa and in the CRCs of those patients expressing the protein (Fig. 9c and d).

## Discussion

CRC is a heterogeneous disease, especially with respect to features such as genetic and dietary interactions and the anatomic location of the tumor [2]. Taking into account the distribution of neoplasia in the colorectum, CRCs are divided into right sided and left sided bowel cancer, and the two types show differences in their clinical presentation and surgical management, as well as functional and molecular differences including the phenotypic expression of various biomarkers [2, 3]. The pathogenesis of CRC usually follows a stepwise progression from benign adenoma to malignant adenocarcinoma and distant metastasis, its sequential nature lending itself to the notion of identifying diagnostic markers.

The expression of HSPGs is markedly altered during malignant transformation and tumor progression, affecting both the PG core proteins and the GAG chains [30]. Substantial evidence in support of this has been reported in relation to various tumors, including brain, breast, lung, skin, pancreas, colon, ovarian and head and neck [30], and as such abnormal HSPG expression in cancer and stromal cells can serve as a biomarker for tumor progression and patient survival [31]. As a result of these modifications, some processes appear to be affected, such as cancer cell signaling, growth and survival, cell adhesion, and differentiation, migration and angiogenesis [31]. The HS fine structure is determined by cell-type specific expression of only certain isoforms of some of the biosynthetic enzymes, notwithstanding the existence in some specific cases of regulation at the level of translation or enzymatic catalysis [32–34]. We have therefore investigated the expression patterns of the genes involved in HSPG biosynthesis in CRCs and compared their expression patterns to that of healthy tissue from the same patients. We have centered the study on right sided tumors due to the fact that there are differences between them and left sided CRCs. In addition, we have considered the comparative analysis of two groups of tumors, both at the T3 stage where the muscularis propria is affected. Also, we included in these analyses, the absence of lymph node metastases.

In human cells, two gene families, syndecans and glypicans, account for most cell surface HSPGs, although a few more part-time proteins may appear [24]. Previous immunohistochemical studies performed with syndecans in colon cancer have demonstrated a loss of expression of syndecan-1 in CRCs, some of which have been found to correlate with tumor stage and metastasis [35–38]. However, a study analyzing mRNA transcripts of syndecan-1 did not find significant differences between normal and cancerous tissues [39], while another, using colon carcinoma cells, evidenced a decrease in syndecan-1 mRNA levels in most of samples, along with a 2–5 fold increase in syndecan-2, and a decrease in syndecan-4 mRNA which was restricted to highly metastatic cell lines [18]. In contrast, in the current work, no significant differences in levels of transcripts of any of the four isoforms were found in non-metastatic CRCs, while syndecan-1 mRNA alone appeared overexpressed in metastatic tumors. Analyzing the expression of syndecan-1 protein using immunohistochemistry, provided the noteworthy finding that non-metastatic CRCs displayed similar immunoreactivity to that detected in normal tissues from the same patients, while the metastatic tumors showed a dramatic decrease in staining. Nevertheless, examining transcription at the tissue level by means of CISH showed positive hybridization in normal as well as tumoral cells, both metastatic and non-metastatic, consistent with the qRT-PCR. These data suggests that in the expression of syndecan-1 in CRCs, additional post-transcriptional mechanisms, such as protein translation, degradation, inhibition by feedback loops or miRNA regulation may be involved. Post-transcriptional regulation of syndecan-1 expression has been indicated previously, for example in pancreatic cancer and peritoneal macrophages [34, 39, 41]. Upregulation of syndecan-1 has also been described in tumors other than those of the colon, and it has been postulated that this aberrant expression may play a key role in promoting growth factor signaling in cancer cells [30]. In contrast, other malignances showed downregulation of this molecule, indicating that this HSPG could well serve as a prognostic marker in a cancer-type-specific manner [30]. Furthermore, at the transcriptional level, syndecan-1 modulation has been related with the expression of other PGs, as well as with genes involved in biosynthesis and sulfation of HS chains [40].

The glypicans constitute a six-member family of cell surface HSPGs that are glycosylphosphatidylinositol-linked to the cell membrane. Their expression is cell-type and developmental-stage specific and they are implicated in the regulation of several signaling pathways, including Wnts, Hedgehogs, FGF, bone morphogenic protein and insulin-like growth factor, where they can either stimulate or inhibit activity depending on the biological context [41]. Because of their biological roles, they influence tumor progression and their abnormal expression has been described in various human tumors [31]. In this work, analysis of the transcription of the 6 genes in CRCs showed deregulations for isoforms 1, 3 and 6 in non-metastatic tumors, while only glypican-1 transcription was significantly altered in metastatic, and to lesser extent than in non-metastatic.

Glypican-1 is, with −3 and −5, the isoform most widely associated with the tumorigenic process. Immunohistochemical analysis of glypican-1 protein showed a very weak staining in most of the cells, although certain specific ones displaying neuroendocrine features showed intense immunostaining, On the other hand, it must be taken into account that the values of transcription quantified by PCR are not probably revealing these differences, since it must be considered that the data are affected by the decrease or absence of neuroendocrine cells with high expression levels in the tumor stroma. The disappearance of the expression of this protein has previously been described in tumor epithelial cells in prostate cancer, although with high expression being maintained in tumor stroma [42]. Conversely, glipican-1 overexpression has been described in certain tumors, although with some peculiarities depending on the neoplasia: in gliomas, where it acts to enhance FGF signaling and mitogenesis [43]; in pancreatic cancer, both in the cancer cells and the adjacent fibroblasts [44]; in high-grade breast cancer tumors [45]. Interestingly, in neuroendocrine neoplasias derived from the large bowel, GPC1 expression has been shown to be intense in well-differentiated tumors, but far less in poorly differentiated, a feature that seems to be shared with other neuroendocrine tumors, independently of their topography [46].

Glypican-3 is though to be the species for which most expression alterations in tumors have been described. In this work, this isoform appeared downregulated in all the non-metastatic CRCs analyzed, while only 50 % of metastatic tumors showed alteration in its level. Downregulation of glypican-3 has been described in many tumor types, including breast, lung, gastric, and ovarian cancers and mesothelioma [41]. However, in tumors originating from tissues where glypican-3 expression is restricted to the embryo, its expression tends to reappear with malignant transformations [41]. The effects of the loss of this protein on tumor development are compatible with its function as a tumor supressor, as this molecule can inhibit cell proliferation and also induces apoptosis [47]. However, GPC3 overexpression can act as an oncogene in some tumors, such as hepatocellular carcinomas [48].

Besides isoforms −1 and −3, 80 % of non-metastatic tumors tested here also displayed a decrease in the transcript levels of glypican-6, a percentage that was halved in CRCs with lymph node metastases. Unlike the other isoforms, relatively little is known concerning the expression or functional roles of glypican-6 in tumors. Reduced expression or loss of function of this isoform has been described in retinoblastoma and autosomal-recessive omodysplasia [49, 50]; however, breast carcinoma invasion has been reported to be promoted through induction of glypican-6 by the transcriptional factor NFAT (nuclear factor of activated T-cells) using non-canonical Wnt5a signaling [51].

In addition to glypicans and syndecans, in this study we also analyzed the expression levels of betaglycan and CD44v3. These molecules are part time membrane HSPGs, that is, they exist either with or without HS chains [24]. CD44 comprises a family of heterogeneous integral membrane PGs derived from a single gene. The HS attachment site is located on exon 8 (CD44v3) [52], and thus we designed primers to specifically detect this isoform. CD44v3 did not show statistically significant differences in any type of CRCs; this data differs from previous studies where the expression of this protein in colon tumors was found to be related to more advanced pathological stage and poorer prognosis [53]; however, further detailed observation of our analysis showed that for 60 % of patients with metastatic tumors, this protein was indeed overexpressed, the scattering of the data having lead to the negative results in the initial statistical analysis. This phenomena was not observed in tumors lacking lymph node metastases, thereby supporting the results obtained in previous studies, which suggests a role for CD44v3 in invasion and metastasis by CRC cells [53].

The other part time HSPG analyzed, betaglycan, appeared underexpressed around 8 fold in non-metastatic CRCs, and about half of the patients with metastatic tumors exhibited under-expression of the protein, a difference that was not statistically significant in this case. Betaglycan is a ubiquitously expressed membrane-bound TGF-β superfamily coreceptor, also known as type III TGF-β receptor, which acts to regulate the cellular actions of TGF-β and inhibin [54]. The expression of betaglycan in tumor cells appears to play an important role in the progression of cancer, and reduced expression of this PG has been associated with advanced stage in different types of cancers [55]. In several neoplastic cells the loss of betaglycan facilitates the epithelial–mesenchymal transition, and a suppressor role for betaglycan in epithelial carcinogenesis has been proposed [55]. However, a correlation between high levels of this PG and invasiveness has been described in breast cancer cell lines [56], and increased expression has been seen in high-grade non-Hodgkin’s lymphomas and B-cell chonic lymphocytic leukemia [57, 58], suggesting a promoting role in these cases.

Two of the three extracellular matrix HSPGs examined here showed significant downregulation in tumoral samples: perlecan and collagen XVIII. Perlecan expression was downregulated at both the mRNA and protein level in tumors, independent of the presence or absence of lymph node metatases. Perlecan is a critical regulator of growth factor-mediated signaling and angiogenesis, and is fundamental for the maintenance of basement membrane homeostasis [59], suggesting that its alteration could play an important role in CRC progression. Although expression of perlecan is enhanced in a number of tumor types, in some other cases, such as lung carcinoma and hepatocellular carcinoma cells, its levels are undetectable and in these cases, it has been suggested that the lack of perlecan may favor the diffusion of growth factors, leading to tumor growth and metastasis [60].

Collagen XVIII transcription levels were also downregulated in CRCs, both metastatic and non-metastatic. Immunohistochemical analysis of the protein revealed that its expression was mainly at arteriolar vessels in the lamina propria in healthy tissues, while in both tumor types its expression could not be detected, although they displayed positive immunostaining using anti-CD34 antibody. CD34 is a cell surface glycoprotein widely used as a marker of vascular endothelial cells [61]. Collagen XVIII expression is widespread throughout vascular basement membranes, and can negatively modulate angiogenic processes by mediating interactions between endothelial cells and underlying extracellular matrix components [62]. The expression of this HSPG has been studied in various malignances and found to vary between different cancer types; increasing in ovarian or pancreatic cancer, while diminishing in liver and oral cancer [31].

Serglycin is the only characterized PG which is located intracellularly, although it has also been documented as a secretory product that may appear incorporated into the ECM or associated with the surface of target cells [25]. In this work, transcript levels of this gene were significantly reduced, both in metastatic and non-metastatic tumors, albeit more intense in the latter. Serglycin is mainly found in hematopoietic and endothelial cells, although some reports suggest it is present in other cell types, such as pancreatic acinar or smooth muscle cells [25, 63]. Nevertheless, in all cell types, the principal GAG chains are CS, except in mast cells where the covalently attached GAG can be CS type E or heparin, depending on their origin [25, 63]. In this study, we performed immunohistochemical analyses using the antibody CD117 to detect mast cells in CRCs. The results showed a drastic reduction in the population of mast cells in tumors compared to non-tumor colon mucosa, a fact which may explain, at least in part, the observed decrease in protein expression. A small number of studies have described alterations in the expression of serglycin in non hematological tumors, such as increased expression in patients with hepatocellular and nasopharingeal carcinoma [64, 65], where it has been related to unfavorable prognosis. Also, its elevated expression in aggressive breast cancer cells has also been reported [66].

The tissue-specific expression of individual HSPGs will determine when and where HS chains are expressed [13]. However, it should be taken into account that some HSPGs can be hybrid molecules, carrying both HS and CS side chains [67]. To generate the GAG chains the regulated expression and action of multiple enzymes, mainly GTs, (which are found in the lumen of the Golgi apparatus) are required [13]. Both HS and CS chain biosynthesis begins with the formation of a tetrasaccharide linkage region that comprises xylose-galactose-galactose-GlcA. Our results revealed that transcript levels of the different enzymes involved in the transfer and modification of the xylose residue display different alterations in CRCs. First, *XYLT1* and *XYLT2* appeared downregulated in non-metastatic patients alone; these genes encode two different xylosyltransferases (XylT1 and XylT2) that transfer a xylose residue from UDP-xylose to the hydroxyl group of a serine on the core protein with differing efficiency and displaying different expression patterns [68]. Modulation of the expression of xylosyltransferases has been described as regulator of GAG-synthesis in rheumatoid conditions [69]. Second, the transcription of *FAM20B*, which encodes xylose kinase 1 that catalyzes a transient phosphorilation of the Xyl residue involved in the control of GAG biosynthesis [70], also decreased significantly, though only in non-metastatic tumors. In relation to the other enzymes involved in the synthesis of the tetrasaccharide linker, only *B3GAT1*, which encodes one of the three isoforms of GlcA-transferase I responsible for the transfer of the GlcA residue [28], appeared downregulated in both types of tumors, although expression was more intense in non-metastatic CRCs.

At this point, the process of GAG synthesis follows one of two divergent paths, depending on whether the addition of a GlcNAc or GalNAc residue takes place, which leads to the synthesis of HS or CS/DS respectively. *EXTL2* and *EXTL3* encode enzymes that possess GlcA-transferase I activity, and thus direct the pathway toward the synthesis of HS, although it has been described that EXTL2 transfer of a GlcNAc residue to a linkage region previously phosphorylated by xylose kinase 1 terminates chain elongation, suggesting that EXTL2 controls HS biosynthesis [70]. The ensuing polymerization of the chain involves the consecutive addition of alternating GlcA and GlcNAc residues, mediated by the action of two enzymes encoded by the genes *EXT1* and *EXT2*. At the level of transcription, the only change found in this set of enzymes was a down-regulation of EXT1 in non-metastatic CRCs. EXT1 and EXT2 are tumor suppressors, associated with hereditary multiple exostoses, characterized by the development of benign skeletal tumors in patients [14]. Although in this study *EXTL3* did not show any significant alterations, previous reports have described its down-regulation in CRCs associated with mucinous differentiation and caused by promoter methylation [21].

The addition of GalNA instead of GlcNAc to the linker directs the pathway towards the biosynthesis of CS. In this case, chain extension takes place through the sequential addition of alternative GlcA and GalNAc residues. Five genes, *CSGALNACT1, CSGALNACT2*, *CHSY1*, *CHPF* and *CHSY3* encode the GTs involved in this process [71]. The expression of all except *CHPF* were found to decrease in the group of non-metastatic tumors analyzed, while in metastatic CRCs, CHPF alone appeared overexpressed. In previous studies of CRCs, a number of changes have been described: a gradual increase in chondroitin polymerizing factor with advancing cancer stage; an increase in CHSY1 expression in normal tissue adjacent to benign tumors compared to tumoral tissues and high expression levels during the early stages; and low expression levels of CHSY3 in both normal and tumor specimens, although it must be stressed that these studies involved colorectal cancers from different locations and not only right sided CRCs [72].

Taken together, the alterations observed in our study in the transcription of this category of genes suggests variation in the GAG chains, particularly in non-metastatic CRCs, while few changes were detected in metastatic tumors. This fact is particularly noticeable in the enzymes involved in the polymerization of CS, which appear to be more affected than those involved in HS polymerization. The decrease of the transcription of some enzymes involved in the synthesis of the tetrasaccharide linker, particularly FAM20B, could also be related. Variations in the level of GAGs for various tumor types have been described elsewhere, both increases and decreases. With respect to CRCs, a decrease in GAG production has been described, as well as reductions in levels of HS [19, 73]. Besides, some studies describe a reduction in CS levels in the neoplastic colon [73], while other reports describe an increase, albeit predominantly related to CS in the intercellular matrix produced by the tissue surrounding the tumor [74].

The fine structure of HS ultimately depends on the control of polymerization of the chains, but the expression and action of multiple sulfotransferases and one epimerase is also essential. As the polymer forms, the first reaction involved in polymer modification involves removal of acetyl groups from GlcNAc residues, followed by sulfation of the amino group which is catalyzed by four different isoforms of N-deacetylase/N-sulfotransferases [13]. NDST1 and NDST2 show broadly overlapping tissue distribution [28], while NDST3 and NDST4 are more restricted and are expressed principally during embryonic development [75]. Our analysis of colon CRCs was able to detect the existence of transcription of isoforms 1 and 2 in both tumoral and normal samples, but not of isoforms 3 and 4. NDST1 and NDST2 appeared significantly underexpressed, but only in non-metastatic tumors. These alterations are likely to involve important consequences for the structure of HS chains, since the reaction catalysed by this family of enzymes is essential for the creating of sulfated S-domains. Lower N-sulfation levels in CRCs involving modifications in both the S domains and their flanking mixed domains have been reported [20, 76], although to our knowledge there are no other studies that compare levels between tumors with or without metastasis.

Further modifications of the HS chains include the epimerization of GlcA into IdoA, catalyzed by the action of the enzyme C5-GlcA epimerase and the addition of O-sulfate groups at C2 of uronic acid and to C6 and C3 of the glucosamine residues [13, 14]. 2-O-sulfation is closely associated with epimerization, and no alteration in the transcription levels of the genes involved, *GLCE* and *HS2ST1*, could be detected in CRCs in this study. O-sulfation at C6 is carried out by enzymes encoded by the genes *HS6ST1*, *HS6ST2* and *HS6ST3*, each of which differs in their substrate specificities and tissue expression [77]. *HS6ST1* transcription here appeared decreased in non-metastatic tumors only; the effect of this downregulation on the sulfation at C6 should be relevant, since *HS6ST1* is the principal isoform, *HS3ST3* levels being found to be very low and to exhibit considerable variation between patients, while *HS6ST2* transcripts were undetected. A reduction in 2-O-sulfation and an increase in 6-O-sulfation in colon carcinoma cells has been described, albeit related to HS from colon adenoma cells [77], and other studies have, in addition, detected the undersulfation of HS molecules in tumors [19].

The final family of enzymes involved in the biosynthesis of HS are the 3-O-sulfotransferases, which in fact form the largest group, comprising seven different members, and which are implicated in the formation of specific HS motifs that interact in a selective manner with specific protein ligands. Although 3-O-sulfation is a relatively rare modification, and to date very few proteins have been described that are influenced by it [78], several studies have described their alteration in different tumors. Such alterations may be upregulations, as in the case of HS3ST1 and HS3ST3A1 in, respectively, hepatocellular cancer and glioblastoma [79, 80], or subexpressions in, for example, HS3ST2 in breast, colon, lung and pancreatic cancers [22], HS3ST1 and HS3ST3A1 in chondrosarcoma cells and HS3ST4, HS3ST5 and HS3ST6 in invasive breast ductal carcinomas [81, 82]. In the present study, isoforms *HS3ST3B1* and *HS3ST5* appeared underexpressed in non-metastatic CRCs. Notwithstanding the implications of 3-O-sulfation decrease in tumors being as yet known, it has been suggested that certain patterns of 3-O-sulfation could impart cancerous phenotypic changes [30].

Of the different sulfate groups present on the HS chains, 6S modification is the only known sulfate moiety known to be post-synthetically edited from the chain, implying that it has special regulatory importance. Two endosulfatases that are secreted from the Golgi and localized on the cell surface, SULF1 and SULF2, selectively remove 6-O-sulfate groups on GlcN residues [27]. Alterations in the expression of these genes have been reported in various tumors, involving either up or downregulations depending on the neoplasia ivolved [17]. However, transcript levels of none of these genes were found to be altered in any type of CRCs.

As indicated earlier, some HSPGs are hybrid molecules, carrying both HS and CS side chains [67]. The alterations observed in this study in transcriptions of GTs largely point towards changes in the CS chains. In addition, changes in CSPGs associated with CRCs, for example versican or decorin, have been described [83]. CS repeating disaccharide building units can be modified by epimerization of GlcA residues and by sulfate groups at C2 of uronic acids and at C4 and/or C6 of GalNAc residues with various combinations [71]. In CRCs, the expressions of the genes encoding the enzymes that catalyze the different reactions which generate CS structures are altered, more than 60 % of them appearing underexpressed. Interestingly, and in contrast to the other groups of genes cited previously, the observed alterations were very similar in both tumor types, irrespective of presence or absence of lymph node metastasis. Furthermore, the observed alterations in expression appear to affect all modifications, except sulfation at C6 of GalNAc, including epimerization and C2 sulfation of uronic acid. In the case of sulfation at C4 of GalNAc, 3 of the 4 genes involved appeared downregulated, the only one showing no significant difference, *CHST12*, being the one which displayed the lowest levels of transcription. In a previous study, the transcriptions of two of these genes, *CHST14* and *CHST3*, were analyzed and it was found that the expression of *CHST3* did not differ, while *CHST14* decreased as the stage of the cancer progressed [76] and, although the samples used were from tumors from different locations, these data support, at least in part, the results presented here. Disaccharide composition analysis carried out in other studies have shown an increase in 6-sulfated and non-sulfated disaccharides, while CS levels were not related with the metastatic potential of a tumor [76, 84]. Interestingly, a recent study described a decrease in the levels of CS in CRC, and this was accompanied by increases in the levels of 6S and 4S6S (CS-E) disaccharide units compared to normal tissue [73]. However, in our study we were not able to detect any transcription of *CHST15*, the gene which encodes the enzyme responsible for transfering sulfate to the C-6 of an already 4-O sulfated GalNAc residue.

HPSE is an endo-β-D-glucuronidase that cleaves specific β-D-glucouronosyl-N-acetyl-glucosaminyl linkages, yielding HS fragments of appreciable size which may contain biologically active HS domains [29]. HPSE expression is induced in all the principal types of human cancer and is often associated with reduced survival, increased tumor metastasis and higher density of microvessels [29]. Analysis of HPSE transcript levels in non-metastatic CRCs in this study showed an apparent decrease in 70 % of the patients analyzed, although the dispersion of values in healthy tissues was considerably higher than in tumoral tissues. In contrast, metastatic tumors showed greater variability in their transcription levels, varying from under to overexpression depending on the individual patient concerned. Conversely, other studies have found HPSE to be expressed at early stages of neoplasia, although it was not detected in the adjacent normal colon epithelium, and its expression gradually increased as the cells progressed from well differentiated to poorly differentiated colon carcinoma [85, 86]. In this study, we were able to detect the expression of HPSE in the adjacent normal-looking colon epithelium, a fact that could be explained by the existence of inflammation [87], or as due to the dynamic constant renewal of the colon mucosa, this latter being a phenomena which has been related to the unmethylated state of the HPSE promoter in the normal colon [88].

HPSE2 is a homologue of heparanase that lacks HS-degrading activity, although it is able to interact with HS with a high affinity, and is capable of modulating HPSE enzymatic activity and signaling properties, to the extent that an anti-metastatic character has been proposed for it [29, 89]. The downregulation of this protein has been described in some neoplasms [82]. In CRCs, transcripts for this gene were detected in 70 % of patients with non-metastatic tumors, which was reduced to 30 % in those cases showing lymph node metastasis, although the dispersion of the data meant that statistically significance was not reached.

## Conclusions

In summary, the analysis of differential expression of the genes involved in the biosynthesis of HSPGs in right sided CRCs indicates that a percentage of them showed significant changes in their transcript levels. The number of genes affected was higher in non-metastatic tumors, involving around 40 % of all genes analyzed, while in metastatic tumors the proportion of genes affected was below 20 %. That said, all genes whose expression was altered in metastatic CRCs also showed altered expression in non-metastatic tumors, the only exceptions being syndecan-1 and *CHPF*. The variations observed seem to have a strong effect on CS chains; in non-metastatic tumors they affect most of the GTs responsible for the polymerization of the saccharide chains as well as having an effect on many enzymes involved in their modification. In contrast, metastatic tumors maintain the alterations in the modifying enzymes, but not those involving the GTs. HS chains seem to experience far more limited changes in metastatic CRCs than in non-metastatic tumors. Moreover, PGs on the cell surface show significant differences in expression depending on the presence or absence of metastases, while alterations observed in proteoglycans located in the extracellular matrix or within the cell are very similar.

## Additional file

Additional file 1: Table S1. qRT-PCR primer sequences. (DOC 253 kb)

## Abbreviations

CRC: Colorectal cancer
CS: Chondroitin sulfate
DS: Dermatan sulfate
ECM: Extracellular matrix
GAG: Glycosaminoglycan
GalNAc: N-acetyl-D-galactosamine
GlcA: D-glucuronic acid
GlcNAc: N-acetyl-D-glucosamine
GT: Glycosyltransferase
HS: Heparan sulfate
HSPG: Heparan sulfate proteoglycan
IdoA: Iduronate

## References

[1] Parkin DM, Bray F, Ferlay J, Pisani P (2005). Global cancer statistics, 2002. CA Cancer J Clin.

[2] Manne U, Shanmugam C, Katkoori VR, Bumpers VR, Grizzle WE (2010). Development and progression of colorectal neoplasia. Cancer Biomark.

[3] Richman S, Adlard J (2002). Left and right sided large bowel cancer. BMJ.

[4] Chau BN, Diaz RL, Saunders MA, Cheng C, Chang AN, Warrener P, Bradshaw J, Linsley PS, Claeary MA (2009). Identification of SULF2 as a novel transcriptional target of p53 by use of integrated genomic analyses. Cancer Res.

[5] Baraz L, Haupt Y, Elkin M, Peretz T, Vlodaysky I (2006). Tumor suppressor p53 regulates heparanase gene expression. Oncogene.

[6] Truant S, Bruyneel E, Gouyer V, De Wever O, Pruvot FR, Mareet M, Huet G (2003). Requirement of both mucins and proteoglycans in cell-cell dissociation and invasiveness of colon carcinoma HT-29 cells. Int J Cancer.

[7] Rider CC (2006). Heparin/heparan sulphate binding in the TGF-beta cytokine superfamily. Biochem Soc Trans.

[8] Lai JP, Oseini AM, Moser CD, Yu C, Elsawa SF, Hu C, Nakamura I, Han T, Aderca I, Isomoto H, Garrity-Park MM, Shire AM, Li J, Sanderson SO, Adjei AA, Fernandez-Zapico ME, Roberts LR (2010). The oncogenic effect of sulfatase 2 in human hepatocellular carcinoma is mediated in part by glypican 3-dependent Wnt activation. Hepatology.

[9] Sakane H, Yamamoto H, Matsumoto S, Sato A, Kikuchi A (2012). Localization of glypican-4 in different membrane microdomains is involved in the regulation of Wnt signaling. J Cell Sci.

[10] Zhao W, McCallum SA, Xiao Z, Zhang F, Linhardt RJ (2012). Binding affinities of vascular endothelial growth factor (VEGF) for heparin-derived oligosaccharides. Biosci Rep.

[11] Mahtouk K, Cremer FW, Rème T, Jourdan M, Baudard M, Moreaux J, Requirand G, Fiol G, De Vos J, Moos M, Quittet P, Goldschmidt H, Rossi JF, Hose D, Klein B (2006). Heparan sulphate proteoglycans are essential for the myeloma cell growth activity of EGF-family ligands in multiple myeloma. Oncogene.

[12] Maglietta R, Liuzzi VC, Cattaneo E, Laczko E, Piepoli A, Panza A, Carella M, Palumbo O, Staiano T, Buffoli F, Andriulli A, Marra G, Ancona N (2012). Molecular pathways undergoing dramatic transcriptomic changes during tumor development in the human colon. BMC Cancer.

[13] Esko JD, Lindahl U (2001). Molecular diversity of heparan sulphate. J Clin Invest.

[14] Whitelock JM, Iozzo RV (2005). Heparan sulfate: a complex polymer charged with biological activity. Chem Rev.

[15] Park PW, Reizes O, Bernfields M (2000). Cell surface heparan sulfate proteoglycans: selective regulators of ligand-receptor encounters. J Biol Chem.

[16] Sanderson RD (2001). Heparan sulfate proteoglycans in invasion and metastasis. Semin Cell Dev Biol.

[17] García-Suárez O, Fernández-Vega I, Quirós LM (2013). Multiple alterations of heparan sulfate in cancer. OA Cancer.

[18] Park H, Kim Y, Lim Y, Han I, Oh ES (2002). Syndecan-2 mediates adhesion and proliferation of colon carcinoma cells. J Biol Chem.

[19] Bouziges F, Simon-Assmann P, Leberquier C, Marescaux J, Bellocq JP, Haffen K, Kedinger M (1990). Changes in glycosaminoglycan synthesis and in heparan sulfate deposition in human colorectal adenocarcinomas. Int J Cancer.

[20] Jayson GC, Lyon M, Paraskeva C, Turnbull JE, Deakin JA, Gallagher JT (1998). Heparan sulfate undergoes specific structural changes during the progression from human colon adenoma to carcinoma in vitro. J Biol Chem.

[21] Karibe T, Fukui H, Sekikawa A, Shiratori K, Fujimori T (2008). EXTL3 promoter methylation down-regulates EXTL3 and heparan sulphate expression in mucinous colorectal cancers. J Pathol.

[22] Miyamoto K, Asada K, Fukutomi T, Okochi E, Yagi Y, Hasegawa T, Asahara T, Sugimura T, Ushijima T (2003). Methylation-associated silencing of heparan sulfate D-glucosaminyl 3-O-sulfotransferase-2 (3-OST-2) in human breast, colon, lung and pancreatic cancers. Oncogene.

[23] Schefe JH, Lehmann KE, Buschmann IR, Unger T, Funke-Kaiser H (2006). Quantitative real-time RT-PCR data analysis: current concepts and the novel “gene expression’s CT difference” formula. J Mol Med.

[24] Sarrazin S, Lamanna WC, Esko JD (2011). Heparan sulfate proteoglycans. Cold Spring Harb Perspect Biol.

[25] Kolset SO, Tveit H (2008). Serglycin-structure and biology. Cell Mol Life Sci.

[26] Carlsson P, Kjellén L (2012). Heparin biosynthesis. Handb Exp Pharmacol.

[27] Kreuger J, Kjellén L (2012). Heparan sulfate biosynthesis: regulation and variability. J Histochem Cytochem.

[28] Nairn AV, Kinoshita-Toyoda A, Toyoda H, Xie J, Harris K, Dalton S, Kulik M, Pierce JM, Toida T, Moremen KW, Linhardt RJ (2007). Glycomics of Proteoglycan Biosynthesis in Murine Embryonic Stem Cell Differentiation. J Proteome Res.

[29] Arvatz G, Shafat I, Levy-Adam F, Ilan N, Vlodavsky I (2011). The heparanase system and tumor metastasis: is heparanase the seed and soil?. Cancer Metast Rev.

[30] Raman K, Kuberan B (2010). Chemical Tumor Biology of Heparan Sulfate Proteoglycans. Curr Chem Biol.

[31] Theocharis A, Skandalis SS, Tzanakakis GN, Karamanos NK (2010). Proteoglycans in health and disease: novel roles for proteoglycans in malignancy and their pharmacological targeting. FEBS J.

[32] Arvatz G, Barash U, Nativ O, Ilan N, Vlodaavsky I (2010). Post-transcriptional regulation of heparanase gene expression by a 3′ AU-rich element. FASEB J.

[33] Grobe K, Esko JD (2002). Regulated translation of heparan sulfate N-acetylglucosamine N-deacetylase/N-sulfotransferase isozymes by structured 5′-untranslated regions and internal ribosome entry sites. J Biol Chem.

[34] Yeaman C, Rapraeger AC (1993). Post-transcriptional regulation of syndecan-1 expression by cAMP in peritoneal macrophages. J Cell Biol.

[35] Day RM, Hao X, Ilyas M (1999). Changes in the expression of syndecan-1 in the colorectal adenoma-carcinoma sequence. Virchows Arch.

[36] Peretti T, Waisberg J, Mader AM, de Matos LL, da Costa RB, Conceicao GM, Lopes AC, Nader HB, Pinhal MA (2008). Heparanase-2, syndecan-1, and extracellular matrix remodeling in colorectal carcinoma. Eur J Gastroenterol Hepatol.

[37] Hashimoto Y, Skacel M, Adams JC (2008). Association of loss of epithelial syndecan-1 with stage and local metastasis of colorectal adenocarcinomas: an immunohistochemical study of clinically annotated tumors. BMC Cancer.

[38] Mitselou A, Skoufi U, Tsimogiannis KE, Briasoulis E, Vougiouklakis T, Arvanitis D, Ioachim E (2012). Association of syndecan-1 with angiogenesis-related markers, extracellular matrix components, and clinicopathological features in colorectal carcinoma. Anticancer Res.

[39] Conejo JR, Kleeff J, Koliopanos A, Matsuda K, Zhu ZW, Goecke H, Bincheng N, Zimmermann A, Korc M, Friess H, Büchler MW (2000). Syndecan-1 expression is up-regulated in pancreatic but not in other gastrointestinal cancers. Int J Cancer.

[40] Szatmári T, Dobra K (2013). The Role of Syndecan-1 in Cellular Signaling and its Effects on Heparan Sulfate Biosynthesis in Mesenchymal Tumors. Front Oncol.

[41] Filmus J, Capurro M, Rast J (2008). Glypicans. Genome Bio.

[42] Suhovskih AV, Mostovich LA, Kunin IS, Boboev MM, Neopmnyashchikh GI, Aidagulova SV, Grigorieva EV (2013). Proteoglycan expression in normal human prostate tissue and prostate cancer. ISRN Oncol.

[43] Su G, Meyer K, Nandini CD, Qiao D, Salamat S, Friedl A (2006). Glypican-1 is frequently overexpressed in human gliomas and enhances FGF-2 signaling in glioma cells. Am J Pathol.

[44] Kleeff J, Ishiwata T, Kumbasar A, Friess H, Büchler MW, Korc M (1998). The cell-surface heparan sulfate proteoglycan glypican-1 regulates growth factor action in pancreatic carcinoma cells and is overexpressed in human pancreatic cancer. J Clin Invest.

[45] Matsuda K, Maruyama H, Guo F, Kleeff J, Itakura J, Matsumoto Y, Lander AD, Korc M (2001). Glypican-1 is overexpressed in human breast cancer and modulates the mitogenic effects of multiple heparin-binding growth factors in breast cancer cells. Cancer Res.

[46] García-Suárez O, García B, Fernández-Vega I, Astudillo A, Quirós LM (2014). Neuroendocrine tumors show altered expresión of chondroitin sulfate, glypican 1, glypican 5, and syndecan 2 depending on their differentiation grade. Front Oncol.

[47] Fico A, Maina F, Dono R (2011). Fine-tuning of cell signalling by glypicans. Cell Mol Life Sci.

[48] Jia HL, Ye QH, Qin LX, Budhu A, Forgues M, Chen Y, Liu YK, Sun HC, Wang L, Lu HZ, Shen F, Tang ZY, Wang XW (2007). Gene expression profiling reveals potential biomarkers of human hepatocellular carcinoma. Clin Cancer Res.

[49] Lau CS, Yu CB, Wong HK, Fan DS, Wong KW, Lam DS, Pang CF, Choy KW (2010). Allelic imbalance at 13q31 is associated with reduced GPC6 in Chinese with sporadic retinoblastoma. Br J Ophthalmol.

[50] Campos-Xavier AB, Martinet D, Bateman J, Belluoccio D, Rowley L, Tan TY, Baxová A, Gustavson KH, Borochowitz ZU, Innes AM, Unger S, Beckmann JS, Mittaz L, Ballhausen D, Superti-Furga A, Savarirayan R, Bonafé L (2009). Mutations in the heparan-sulfate proteoglycan glypican 6 (GPC6) impair endochondral ossification and cause recessive omodysplasia. Am J Hum Genet.

[51] Yiu GK, Kaunisto A, Chin YR, Toker A (2011). NFAT promotes carcinoma invasive migration through glypican-6. Biochem J.

[52] Cooper DL, Dougherty GJ (1995). To metastasize or not? Selection of CD44 splice sites. Nat Med.

[53] Kuniyasu H, Oue N, Tsutsumi M, Tahara E, Yasui W (2001). Heparan sulfate enhances invasion by human colon carcinoma cell lines through expression of CD44 variant exon 3. Clin Cancer Res.

[54] Hempel N, How T, Dong M, Murphy S, Fileds TA, Blobe GC (2007). Loss of betaglycan expression in ovarian cancer: role in motility and invasión. Cancer Res.

[55] Bernabeu C, Lopez-Novoa JM, Quintanilla M (2009). The emerging role of TGF-beta superfamily coreceptors in cancer. Biochim Biophys Acta.

[56] Criswell TL, Dumont N, Barnett JV, Arteaga CL (2008). Knockdown of the transforming growth factor-beta type III receptor impairs motility and invasion of metastatic cancer cells. Cancer Res.

[57] Woszczyk D, Gola J, Jurzak M, Mazurek U, Mykata-Ciesla J, Wilczok T (2004). Expression of TGF beta1 genes and their receptor types I, II, and III in low- and high-grade malignancy non-Hodgkin’s lymphomas. Med Sci Monit.

[58] Jelinek DF, Tschumper RC, Stolovitzky GA, Iturria SJ, Tu Y, Lepre J, Shah N, Kay NE (2003). Identification of a global gene expression signature of B-chronic lymphocytic leucemia. Mol Cancer Res.

[59] Iozzo RV, Zoeller JJ, Nyström A (2009). Basement membrane proteoglycans: modulators Par Excellence of cancer growth and angiogenesis. Mol Cells.

[60] Xinnong J, Couchman J (2003). Perlecan and tumor angiogenesis. J Histochem Cytochem.

[61] Nielsen JS, McNagny KM (2008). Novel functions of the CD34 family. J Cell Sci.

[62] Li Q, Olsen BR (2004). Increased angiogenic response in aortic explants of collagen XVIII/endostatin-null mice. Am J Pathol.

[63] Korpetinou A, Skandalis SS, Labropoulou VT, Smirlaki G, Noulas A, Karamanos NK, Theocharis AD (2014). Serglycin: At the Crossroad of Inflammation and Malignancy. Front Oncol.

[64] He L, Zhou X, Qu C, Tang Y, Zhang Q, Hong J (2013). Serglycin (SRGN) overexpression predicts poor prognosis in hepatocellular carcinoma patients. Med Oncol.

[65] Li XJ, Ong CK, Cao Y, Xiang YQ, Shao JY, Ooi A, Peng LX, Lu WH, Zhang Z, Petillo D, Qin L, Bao YN, Zheng FJ, Chia CS, Iyer NG, Kang TB, Zeng YX, Soo KC, Trent JM, Teh BT, Qian CN (2011). Serglycin is a theranostic target in nasopharyngeal carcinoma that promotes metastasis. Cancer Res.

[66] Korpetinou A, Skandalis SS, Moustakas A, Happonen KE, Tveit H, Prydz K, Labropoulou VT, Giannopoulou E, Kalofonos HP, Blom AM, Karamanos NK, Theocharis AD (2013). Serglycin is implicated in the promotion of aggressive phenotype of breast cancer cells. PLoS One.

[67] Iozzo RV (2001). Heparan sulfate proteoglycans: intrincate molecules with intriguing functions. J Clin Invest.

[68] Pönighaus C, Ambrosius M, Casanova JC, Prante C, Kuhn J, Esko JD, Kleesiek K, Götting C (2007). Human xylosyltransferase II is involved in the biosynthesis of the uniform tetrasaccharide linkage region in chondroitin sulphate and heparan sulphate proteoglycans. J Biol Chem.

[69] Venkatesan N, Barré L, Magdalou J, Mainard D, Netter P, Fournel-Gigleux S, Ouzzine M (2009). Modulation of xylosyltransferase I expression provides a mechanism regulating glycosaminoglycan chain synthesis during cartilage destruction and repair. FASEB J.

[70] Nadanaka S, Zhou S, Kagiyama S, Shoji N, Sugahara K, Sugihara K, Asano M, Kitagawa H (2013). EXTL2, a member of EXT family of tumor suppressor, controls glycosaminoglycan biosynthesis in a xylose kinase-dependent manner. J Biol Chem.

[71] Mizumoto S, Ikegawa S, Sugahara K (2013). Human genetic disorders caused by mutations in genes encoding biosynthetic enzymes for sulfated glycosaminoglycans. J Biol Chem.

[72] Kalathas D, Theocharis DA, Bounias D (2011). Chondroitin synthases I, II, III and chondroitin sulfate glucuronyltransferase expression in colorectal cancer. Mol Med Rep.

[73] Joo EJ, Weyers A, Li G, Gasimli L, Li L, Choi WJ, Lee KB, Linhardt RJ (2014). Carbohydrate-Containing Molecules as Potential Biomarkers in Colon Cancer. OMICS.

[74] Iozzo RV, Bolender RP, Wight TN (1982). Proteoglycan changes in the intercellular matrix of human colon carcinoma: an integrated biochemical and stereologic analysis. Lab Invest.

[75] Grobe K, Ledin J, Ringvall M, Holmborn K, Forsberg E, Esko JD, Kjellén L (2002). Heparan sulfate and development: differential roles of the N-acetylglucosamine N-deacetylase/N-sulfotransferase isozymes. Biochim Biophys Acta.

[76] Kalathas D, Theocharis DA, Bounias D, Kyriakopoulou D, Papageorgakopoulou N, Stavropoulos MS, Vynios DH (2009). Alterations of glycosaminoglycan disaccharide content and composition in colorectal cancer: structural and expressional Studies. Oncol Rep.

[77] Smeds E, Habuchi H, Do AT, Hjertson E, Grundberg H, Kimata K, Lindahl U, Kusche-Gullberg M (2003). Substrate specificities of mouse heparan sulphate glucosaminyl 6-O-sulphotransferases. Biochem J.

[78] Thacker BE, Xu D, Lawrence R, Esko JD (2014). Heparan sulfate 3-O-sulfation: A rare modification in search of a function. Matrix Biol.

[79] Tátrai P, Egedi K, Somorácz A, van Kuppevelt TH, Ten Dam G, Lyon M, Deakin JA, Kiss A, Schaff Z, Kovalszky I (2010). Quantitative and qualitative alterations of heparan sulphate in fibrogenic liver diseases and hepatocellular cancers. J Histochem Cytochem.

[80] Wade A, Robinson AE, Engler JR, Petrisch C, James CD, Phillips JJ (2013). Proteoglycans and their roles in brain cancer. FEBS J.

[81] Bui C, Ouzzine M, Talhaoui I, Sharp S, Prydz K, Coughtrie MW, Fournel-Gigleux S (2010). Epigenetics: methylation-associated repression of heparan sulfate 3-O-sulfotransferase gene expression contributes to the invasive phenotype of H-EMC-SS chondrosarcoma cells. FASEB J.

[82] Fernández-Vega I, García O, Crespo A, Castañón S, Menéndez P, Astudillo A, Quirós LM (2013). Specific genes involved in synthesis and editing of heparan sulphate proteoglycans show altered expression patterns in breast cancer. BMC Cancer.

[83] Asimakopoulou AP, Theocharis AD, Tzanakakis GN, Karamanos NK (2008). The biological role of chondroitin sulfate in cancer and chondroitin-based anticancer agents. In Vivo.

[84] Theocharis AD, Theocharis DA (2002). High-performance capillary electrophoretic analysis of hyaluronan and galactosaminoglycan-disaccharides in gastrointestinal carcinomas. Differential disaccharide composition as a possible tool-indicator for malignancies. Biomed Chromatogr.

[85] Friedmann Y, Vlodavsky I, Aingorn H, Aviv A, Peretz T, Pecker I, Pappo O (2000). Expression of heparanase in normal, dysplastic, and neoplastic human colonic mucosa and stroma. Evidence for its role in colonic tumorigenesis. Am J Pathol.

[86] Nobuhisa T, Naomoto Y, Ohkawa T, Takaoka M, Ono R, Murata T, Gunduz M, Shirakawa Y, Yamatsuji T, Haisa M, Matsuoka J, Tsujigiwa H, Nagatsuka H, Nakajima M, Tanaka N (2005). Heparanase expression correlates with malignant potential in human colon cancer. J Cancer Res Clin Oncol.

[87] Hermano E, Lerner I, Elkin M (2012). Heparanase enzyme in chronic inflammatory bowel disease and colon cancer. Cell Mol Life Sci.

[88] Peerless Y, Simon E, Sabo E, Ben-Izhak O, Hershkovitz D (2013). Normal colon tissue and colon carcinoma show no difference in heparanase promoter methylation. Exp Mol Pathol.

[89] Levy-Adam F, Feld S, Cohen-Kaplan V, Shteingauz A, Gross M, Arvatz G, Naroditsky I, Ilan N, Doweck I, Vlodavsky I (2010). Heparanase 2 interacts with heparan sulfate with high affinity and inhibits heparanase activity. J Biol Chem.

