# Supplementary material for: Heparan sulfate proteoglycans undergo differential expression alterations in right sided colorectal cancer, depending on their metastatic character
Source: BMC Cancer. 2015 Oct 20;15:742. doi: 10.1186/s12885-015-1724-9 (PMC4617710; doi:10.1186/s12885-015-1724-9)
Supplement: Additional file 1: Table S1. — qRT-PCR primer sequences. (DOC 253 kb) [file 12885_2015_1724_MOESM1_ESM.doc]

**Additional file 1**

**Table S1**: qRT-PCR primer sequences

| **Gene** | **Gene ID** | **Primer sequence** |
| --- | --- | --- |
| SDC1 | [6382](http://www.ncbi.nlm.nih.gov/sites/entrez?cmd=search&db=gene&term=6382%5Buid%5D) | F 5’- CTCAGGTGCAGGTGCTTTG  R 5’- CTGCGTGTCCTTCCAAGTG |
| SDC2 | [6383](http://www.ncbi.nlm.nih.gov/sites/entrez?cmd=search&db=gene&term=6383%5Buid%5D) | F 5’- GATGACGATGACTACGCTTCTG  R 5’-TGGAAGTGGTCGAGATGTTG |
| SDC3 | [9672](http://www.ncbi.nlm.nih.gov/sites/entrez?cmd=search&db=gene&term=9672%5Buid%5D) | F 5’- CTCCTTTCCCGATGATGAAC  R 5’-CGACTCCTGCTCGAAGTAGC |
| SDC4 | [6385](http://www.ncbi.nlm.nih.gov/sites/entrez?cmd=search&db=gene&term=6385%5Buid%5D) | F 5’-GGCAGGAATCTGATGACTTTG  R 5’-TCTAGAGGCACCAAGGGATG |
| GPC1 | [2817](http://www.ncbi.nlm.nih.gov/sites/entrez?cmd=search&db=gene&term=2817%5Buid%5D) | F 5’-CATCGGGTGTGGAGAGTG  R 5’-TGAGCGTGTCCCTGTTGTC |
| GPC2 | [221914](http://www.ncbi.nlm.nih.gov/sites/entrez?cmd=search&db=gene&term=221914%5Buid%5D) | F 5’-CTGGGACACGACCTGGAC  R 5’-GCCATCCAGTCATCTGCATAC |
| GPC3 | [2719](http://www.ncbi.nlm.nih.gov/sites/entrez?cmd=search&db=gene&term=2719%5Buid%5D) | F 5’-CTGCTTCAGTCTGCAAGTATGG  R 5’-GTGGAGTCAGGCTTGGGTAG |
| GPC4 | [2239](http://www.ncbi.nlm.nih.gov/sites/entrez?cmd=search&db=gene&term=2239%5Buid%5D) | F 5’-AGTGTGGTCAGCGAACAGTG  R 5’-CAAACATATCATTCAGGGATTTCTC |
| GPC5 | [2262](http://www.ncbi.nlm.nih.gov/sites/entrez?cmd=search&db=gene&term=2262%5Buid%5D) | F 5’-GCCGCCCTGTAAGAACAC  R 5’-TCATTCCATGCTTCTCTTTGC |
| GPC6 | [10082](http://www.ncbi.nlm.nih.gov/sites/entrez?cmd=search&db=gene&term=10082%5Buid%5D) | F 5’-CCAGGCATAAGAAATTTGACG  R 5’-CATGTACAGCATGCCATAGGTC |
| PRCAN | 3339 | F 5’-TGGACACATTCGTACCTTTCTG  R 5’-CACTGCCCAGGTCGTCTC |
| AGRN | [375790](http://www.ncbi.nlm.nih.gov/sites/entrez?cmd=search&db=gene&term=375790%5Buid%5D) | F 5’-ACTGTGTCTGCCCGATGC  R 5’- GACACTCGTTGCCGTATGTG |
| COL18A1 | [80781](http://www.ncbi.nlm.nih.gov/sites/entrez?cmd=search&db=gene&term=80781%5Buid%5D) | F 5’-GTACAAGGGAGAGATTGGCTTTC  R 5’- TTTCTCTCCTTTCAATCCGTTC |
| TGFBR3 | 7049 | F 5’- agtgtgagctgacgctgtgta  R 5’- gggcttagtgaacgtcttcttattc |
| CD44 (v3) | 960 | F 5’- TGGGAGCCAAATGAAGAAAATGAA  R 5’- TGGTTGAAATGGTGCTGGAGA |
| SRGN | 5552 | F 5’- TCCTGGTTCTGGAATCCTCA  R 5’- TCTTGTTGGATTCACCTGGAA |
| XYLT1 | 64131 | F 5’- ACTACCCCATCAGGACAAATGA  R 5’- CTGCTTCCGAATGAACCTTG |
| XYLT2 | 64132 | F 5’- AGGGCCTGGTAGTGTGGAG  R 5’- TGAACTGTCTGTGTCCTTGGAA |
| FAM20B | 9917 | F 5’- Tctgcagaagcaccgtca  R 5’- cagctgtgtcaatgatgtcca |
| B4GALT7 | 11285 | F 5’- gcgaggacgacgagttctac  R 5’- Caggtggcgaaatgtcttgta |
| B3GALT6 | 126792 | F 5’- cacgtggccttcgagttc  R 5’- ccgagaagaagccccagta |
| B3GAT1 | 27087 | F 5’- tggtgaatgagggcaagaa  R 5’- cttaggagtcggccttgga |
| B3GAT2 | 135152 | F 5’- gctgacgacgacaacaccta  R 5’- cggtgtaccagccaacaac |
| B3GAT3 | [26229](http://www.ncbi.nlm.nih.gov/sites/entrez?cmd=search&db=gene&term=26229%5Buid%5D) | F 5’-GAAGAACGTGTTTCTCGCCTAC  R 5’- CCTCAGATCCTTCTGCCGTA |
| EXTL2 | [2135](http://www.ncbi.nlm.nih.gov/sites/entrez?cmd=search&db=gene&term=2135%5Buid%5D) | F 5’-TGAACTGGAAACCAATGCAG  R 5’- AGGAAATTGCTGCCAAACTG |
| EXTL3 | 2137 | F 5’- Ctccgccatgacgaaatc  R 5’- agttggagttgtagagccagga |
| EXT1 | [2131](http://www.ncbi.nlm.nih.gov/sites/entrez?cmd=search&db=gene&term=2131%5Buid%5D) | F 5’-GAGACAATGATGGGACAGACTTC  R 5’-CTCTGTCGCTGGGCAAAG |
| EXT2 | [2132](http://www.ncbi.nlm.nih.gov/sites/entrez?cmd=search&db=gene&term=2132%5Buid%5D) | F 5’-CTGGGACCATGAGATGAATA  R 5’- GATATCCCCAGGCATTTTGTA |
| CSGALNACT1 | [55790](http://www.ncbi.nlm.nih.gov/sites/entrez?cmd=search&db=gene&term=55790%5Buid%5D) | F 5’-ggagaccctgaacaatcctg  R 5’- gccgtttgaattcgtgtttg |
| CSGALNACT2 | 55454 | F 5’- gccattgtttatgccaacca  R 5’- atccaccaatggtcaggaaa |
| CHSY1 | [22856](http://www.ncbi.nlm.nih.gov/sites/entrez?cmd=search&db=gene&term=22856%5Buid%5D) | F 5’-GCCCAGAAATACCTGCAGAC  R 5’-GCACTACTGGAATTGGTACAGATG |
| CHPF | [79586](http://www.ncbi.nlm.nih.gov/sites/entrez?cmd=search&db=gene&term=79586%5Buid%5D) | F 5’-GGTGCACTATAGCCATCTGGA  R 5’- GGCACTTCGGAAATGAGG |
| CHSY3 | [337876](http://www.ncbi.nlm.nih.gov/sites/entrez?cmd=search&db=gene&term=337876%5Buid%5D) | F 5’-GACTCAGTGTGTCTGGTCTTACG  R 5’- TTGCTATTGTGAAGGTCTTGGA |
| NDST1 | [3340](http://www.ncbi.nlm.nih.gov/sites/entrez?cmd=search&db=gene&term=3340%5Buid%5D) | F 5’-CTGCCCTCTACCTGTTCCTG  R 5’- AACTGGATCTCCTCAAAGGTCTC |
| NDST2 | [8509](http://www.ncbi.nlm.nih.gov/sites/entrez?cmd=search&db=gene&term=8509%5Buid%5D) | F 5’-CAAGAGCTGCGTACCAACC  R 5’-GAGGGTCCGTGTGTAGTTCAG |
| NDST3 | [9348](http://www.ncbi.nlm.nih.gov/sites/entrez?cmd=search&db=gene&term=9348%5Buid%5D) | F 5’-CCTTGCAGAAGAGATGTTTGG  R 5’- GTAGCAGGATCAGTTCTTAGTTGTTG |
| NDST4 | [64579](http://www.ncbi.nlm.nih.gov/sites/entrez?cmd=search&db=gene&term=64579%5Buid%5D) | F 5’-GACATTGGGCTCCATCTGAC  R 5’- GCTGCTGTCCATCAATAATTAGC |
| GLCE | [26035](http://www.ncbi.nlm.nih.gov/sites/entrez?cmd=search&db=gene&term=26035%5Buid%5D) | F 5’-TGTGGAAGTCCGAGACAGAG  R 5’- CTGGATTGGATAGAAATAGCCTTG |
| HS2ST1 | [9653](http://www.ncbi.nlm.nih.gov/sites/entrez?cmd=search&db=gene&term=9653%5Buid%5D) | F 5’-TGGAGATGATTATAGACCAGGGTTAC  R 5’-GCTATGGCCACAGAAGAACG |
| HS6ST1 | [9394](http://www.ncbi.nlm.nih.gov/sites/entrez?cmd=search&db=gene&term=9394%5Buid%5D) | F 5’-Gcagggagtggagctaacag  R 5’- aacagttccagttcccgaaa |
| HS6ST2 | [90161](http://www.ncbi.nlm.nih.gov/sites/entrez?cmd=search&db=gene&term=90161%5Buid%5D) | F 5’-Cggtgcgatcttctccaa  R 5’-aggacgatcacggcaaatag |
| HS6ST3 | [266722](http://www.ncbi.nlm.nih.gov/sites/entrez?cmd=search&db=gene&term=266722%5Buid%5D) | F 5’-CAACCACAGCCACACCAG  R 5’- CTTCTTCCATCACACATATGAAGAG |
| HS3ST1 | [9957](http://www.ncbi.nlm.nih.gov/sites/entrez?cmd=search&db=gene&term=9957%5Buid%5D) | F 5’-CAGCCAGATGCCCTTCTC  R 5’- AGACTCGCTCAGGCACTTTG |
| HS3ST2 | [9956](http://www.ncbi.nlm.nih.gov/sites/entrez?cmd=search&db=gene&term=9956%5Buid%5D) | F 5’-GATTGGTACAGGAGCCTGATG  R 5’- GGAGCCTCTTGAGTGACAAAG |
| HS3ST3A1 | 9955 | F 5’-ggccgagagaacctgaactc  R 5’- cgagcgacagtgacttcca |
| HS3ST3B1 | 9953 | F 5’- gcagatcttgcctcgatgtc  R 5’- gcgcacgagtacaggaacata |
| HS3ST4 | [9951](http://www.ncbi.nlm.nih.gov/sites/entrez?cmd=search&db=gene&term=9951%5Buid%5D) | F 5’-TAGAGCCGCACTTCTTCGAC  R 5’-GGTTATTTGCCCATCCAAAG |
| HS3ST5 | [222537](http://www.ncbi.nlm.nih.gov/sites/entrez?cmd=search&db=gene&term=222537%5Buid%5D) | F 5’-CATCCGGCAGTAGTCAAAGC  R 5’- TTGTGATTTGCTGAGGGTAGG |
| HS3ST6 | [64711](http://www.ncbi.nlm.nih.gov/sites/entrez?cmd=search&db=gene&term=64711%5Buid%5D) | F 5’-GCCCTGCTGGAGTTTCTG  R 5’- GCGCTCGTAGCACCTGTC |
| SULF1 | [23213](http://www.ncbi.nlm.nih.gov/sites/entrez?cmd=search&db=gene&term=23213%5Buid%5D) | F 5’-CCAGCAGAAGCCAAAGAAAG  R 5’- GAACGTGTCTGCCGAGTATG |
| SULF2 | [55959](http://www.ncbi.nlm.nih.gov/sites/entrez?cmd=search&db=gene&term=55959%5Buid%5D) | F 5’-GCCTGCAAGAGAAGGACAAG  R 5’-AGCAGCTTGCGGAGTTTC |
| CHST11 | [50515](http://www.ncbi.nlm.nih.gov/sites/entrez?cmd=search&db=gene&term=50515%5Buid%5D) | F 5’-CGCTGCTGGAAGTGATGA  R 5’- AGGATAAAGGATCCCAAGCAA |
| CHST12 | [55501](http://www.ncbi.nlm.nih.gov/sites/entrez?cmd=search&db=gene&term=55501%5Buid%5D) | F 5’-GTAGCCGACAAATCCTTCCA  R 5’- ACCGGTTTACCTCTGACTTGAC |
| CHST13 | [166012](http://www.ncbi.nlm.nih.gov/sites/entrez?cmd=search&db=gene&term=166012%5Buid%5D) | F 5’-CCGGCATTTGGAAACAGA  R 5’- TCCAGGTCATAGAGCTTCTGC |
| CHST14 | [113189](http://www.ncbi.nlm.nih.gov/sites/entrez?cmd=search&db=gene&term=113189%5Buid%5D) | F 5’-ccactgcctaatgtcaccaa  R 5’- ATGACAGGCAGAAGCACAGA |
| CHST15 | [51363](http://www.ncbi.nlm.nih.gov/sites/entrez?cmd=search&db=gene&term=51363%5Buid%5D) | F 5’-Gtgccaggaataaagttcaaca  R 5’- cactggataagtcccgagtga |
| CHST3 | [9469](http://www.ncbi.nlm.nih.gov/sites/entrez?cmd=search&db=gene&term=9469%5Buid%5D) | F 5’-TGCACAGCCTGAAGATGAGA  R 5’- CAGCTTGTCTGAGACCCTTGA |
| CHST7 | [56548](http://www.ncbi.nlm.nih.gov/sites/entrez?cmd=search&db=gene&term=56548%5Buid%5D) | F 5’-GATCCGGGTCAGTCACCA  R 5’-GACAGATTGCCCCCACAG |
| DSE | [29940](http://www.ncbi.nlm.nih.gov/sites/entrez?cmd=search&db=gene&term=29940%5Buid%5D) | F 5’-GTCCAGAGGCACTTCAACATC  R 5’- AGTCCGCAATAGCCACAGTC |
| UST | [10090](http://www.ncbi.nlm.nih.gov/sites/entrez?cmd=search&db=gene&term=10090%5Buid%5D) | F 5’-ACCATGGACCACCTCCTAGTAA  R 5’- CACACTTGCCTACCCTGTTGTA |
| HPSE | [10855](http://www.ncbi.nlm.nih.gov/sites/entrez?cmd=search&db=gene&term=10855%5Buid%5D) | F 5’-ATGCTCAGTTGCTCCTGGAC  R 5’- CTCCTAACTGCGACCCATTG |
| HPSE2 | [60495](http://www.ncbi.nlm.nih.gov/sites/entrez?cmd=search&db=gene&term=60495%5Buid%5D) | F 5’-caccctgatgttatgctggag  R 5’- tccagagcaatcagcaaagtta |
